# Reciprocal Prioritization to Dietary Glycans by Gut Bacteria in a Competitive Environment Promotes Stable Coexistence

**DOI:** 10.1128/mBio.01068-17

**Published:** 2017-10-10

**Authors:** Yunus E. Tuncil, Yao Xiao, Nathan T. Porter, Bradley L. Reuhs, Eric C. Martens, Bruce R. Hamaker

**Affiliations:** a Whistler Center for Carbohydrate Research, Food Science Department, Purdue University, West Lafayette, Indiana, USA; b Department of Microbiology and Immunology, University of Michigan, Ann Arbor, Michigan, USA; University of Alberta; University of Hawaii at Manoa

**Keywords:** carbohydrate utilization, hierarchical preference, microbiota, polysaccharide utilization loci, transcription

## Abstract

When presented with nutrient mixtures, several human gut *Bacteroides* species exhibit hierarchical utilization of glycans through a phenomenon that resembles catabolite repression. However, it is unclear how closely these observed physiological changes, often measured by altered transcription of glycan utilization genes, mirror actual glycan depletion. To understand the glycan prioritization strategies of two closely related human gut symbionts, *Bacteroides ovatus* and *Bacteroides thetaiotaomicron*, we performed a series of time course assays in which both species were individually grown in a medium with six different glycans that both species can degrade. Disappearance of the substrates and transcription of the corresponding polysaccharide utilization loci (PULs) were measured. Each species utilized some glycans before others, but with different priorities per species, providing insight into species-specific hierarchical preferences. In general, the presence of highly prioritized glycans repressed transcription of genes involved in utilizing lower-priority nutrients. However, transcriptional sensitivity to some glycans varied relative to the residual concentration in the medium, with some PULs that target high-priority substrates remaining highly expressed even after their target glycan had been mostly depleted. Coculturing of these organisms in the same mixture showed that the hierarchical orders generally remained the same, promoting stable coexistence. Polymer length was found to be a contributing factor for glycan utilization, thereby affecting its place in the hierarchy. Our findings not only elucidate how *B. ovatus* and *B. thetaiotaomicron* strategically access glycans to maintain coexistence but also support the prioritization of carbohydrate utilization based on carbohydrate structure, advancing our understanding of the relationships between diet and the gut microbiome.

## IMPORTANCE

The microorganisms that reside in the human colon fulfill their energy requirements mainly from diet- and host-derived complex carbohydrates. Members of this ecosystem possess poorly understood strategies to prioritize and compete for these nutrients. Based on direct carbohydrate measurements and corresponding transcriptional analyses, our findings showed that individual bacterial species exhibit different preferences for the same set of glycans and that this prioritization is maintained in a competitive environment, which may promote stable coexistence. Such understanding of gut bacterial glycan utilization will be essential to eliciting predictable changes in the gut microbiota to improve health through the diet.

## INTRODUCTION

The majority of dietary polysaccharides (glycans), with the exception of starch, are not degraded and absorbed in the small intestine due to the lack of corresponding enzymes for their digestion. These fiber glycans reach the colon, where they are utilized by the community of hundreds of different microbial species (collectively known as the microbiota). Through glycan fermentation, gut microbes generate biologically important compounds, such as short-chain fatty acids (SCFAs), which benefit the host by contributing to the host’s daily caloric requirement (1), improving gut epithelial health and immune system development, and exerting anticarcinogenic effects (2–4).

Indigestible dietary glycans are important in determining gut microbial community composition because these nutrients disproportionately fill the growth requirements of the microbiota vis-à-vis other nutrient groups (fats, proteins, starch) that are not degraded and absorbed in the proximal intestine. Consumption of different types of glycans or a fiber-rich diet favors different bacteria or bacterial groups in the colon (5–11), and these effects may occur in less than 24 h (12). This shift in composition can be attributed to the differences in the genetically encoded glycan-degrading abilities of various species, which favor some glycans while disfavoring others, based on optimal utilization of particular carbohydrates (5).

The human colonic microbiota is dominated by members of only a few phyla, with *Firmicutes* and *Bacteroidetes* generally the most abundant (13–15). The high abundance of members of the *Bacteroidetes* phylum, especially the genus *Bacteroides*, is attributed to their ability to catabolize a broad range of complex carbohydrates as well as host-derived glycans (11, 16). Glycan utilization by members of the *Bacteroides* species is typically mediated by gene clusters termed polysaccharide utilization loci (PULs) (17, 18). Each PUL encodes carbohydrate-binding proteins, transporters, and enzymes that target a particular glycan for breakdown (18, 19). *Bacteroides* species possess cognate PULs for all glycans that they are capable of degrading (16, 20, 21), and expression of a given PUL is activated by either an oligosaccharide derived from a larger glycan (21) or its monosaccharide units (5).

Most diets, even those represented by just one dietary fiber source, contain more than one type of glycan, and utilization of these different structures by gut bacteria requires activation of multiple PULs. For example, pectins from various botanical sources may be composed of arabinan (ARAB), arabinogalactan, homogalacturonan, pectic galactan (PG), and rhamnogalacturonans I and II (RGI and RGII). *Bacteroides thetaiotaomicron* expresses at least 8 PULs to utilize just these pectic structures (21). This has raised the question of whether *Bacteroides* species activate all of their PULs simultaneously to utilize different glycans found as a mixture or if they activate them in a prioritized order. When grown in mixtures containing up to 12 different glycans, *B. thetaiotaomicron* was shown to preferentially utilize certain glycans before others (20, 22–25). Thus, even though many colonic bacterial species have the ability to utilize a number of glycans, they do not simultaneously deploy this ability against all these glycans and instead resort to prioritized utilization of these nutrients (24). This metabolic hierarchy is species specific, with different species exhibiting variable and sometimes opposite rank orders for some glycans (25). A mechanistic understanding of such preferences has been partially revealed for *B. thetaiotaomicron* (25); monosaccharides derived from degradation of certain glycans repress expression of PULs that target low-priority mucin *O*-linked glycans through the action of a *cis*-acting intergenic region of the PUL transcript. Additional pieces of local and global PUL regulatory mechanisms have been discovered and implicate a global regulator with homology to the *Escherichia coli* catabolite repression protein (CRP), alterations in phosphorylation of locally acting positive transcription factors, and also *cis*-encoded small antisense RNAs (26–29).

Each of these studies relied on monitoring changes in PUL transcription or microbial behavior, such as diauxic growth. However, none has directly monitored changes in the amounts of residual glycans, which may be important since gene expression does not necessarily directly correlate with glycan utilization, as the abundance and activity of enzymes might be further controlled by posttranscriptional events.

In this study, we addressed the question of whether glycan depletion tracks directly with transcriptional regulation of genes involved in glycan utilization. We further questioned whether hierarchical preferences are retained in a competitive environment, which has important implications for understanding the biological contributions of these behaviors to gut colonization. To answer these questions, we performed a series of *in vitro* experiments using two closely related *Bacteroides* species, *B. ovatus* and *B. thetaiotaomicron*, which are both commonly found in the gut of adult humans (15). Based on direct glycan depletion data, which can be considered benchmarks to assess utilization, these closely related species exhibited different hierarchical preferences for glycans presented as a mixture, and transcript levels were not always found to correlate with glycan abundance. Glycan utilization hierarchies remained intact even when the bacteria were cocultured, providing further support for the idea that these behaviors are “hardwired” and may not be subject to alterations in microbial communities. Similarly, while the molecular structure of a glycan (polymer length) was found to contribute to its recognition and utilization by bacteria, an extreme change in this parameter only altered preferential use slightly, indicating that other factors, such as the identities of sugars contained in cometabolized glycans, are dominant in these responses. Taken together, our results provide further knowledge about the relationship between glycan utilization and the colonic microbiota, which will ultimately assist in designing strategies for the predictable manipulation of the colonic microbiota.

## RESULTS

### Direct depletion of glycans from complex mixtures differs from changes in transcriptional activity.

**FIG 1:**
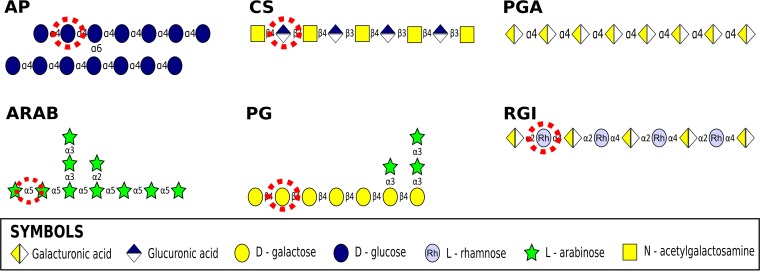
Chemical structures of glycans used. Dashed circles indicate the monosaccharides or linkages that were used to measure the remaining amount of corresponding glycan in media throughout experiments. Remaining amounts of AP, CS, RGI, and PG relative to their initial amounts were determined by measuring the glucose, glucuronic acid, rhamnose, and galactose amounts in the samples, respectively. The remaining ARAB amount was measured by quantifying the remaining 5-arabinose linkage over time. The remaining PGA amount was calculated by subtracting total rhamnose from total galacturonic acid.

**FIG 2:**
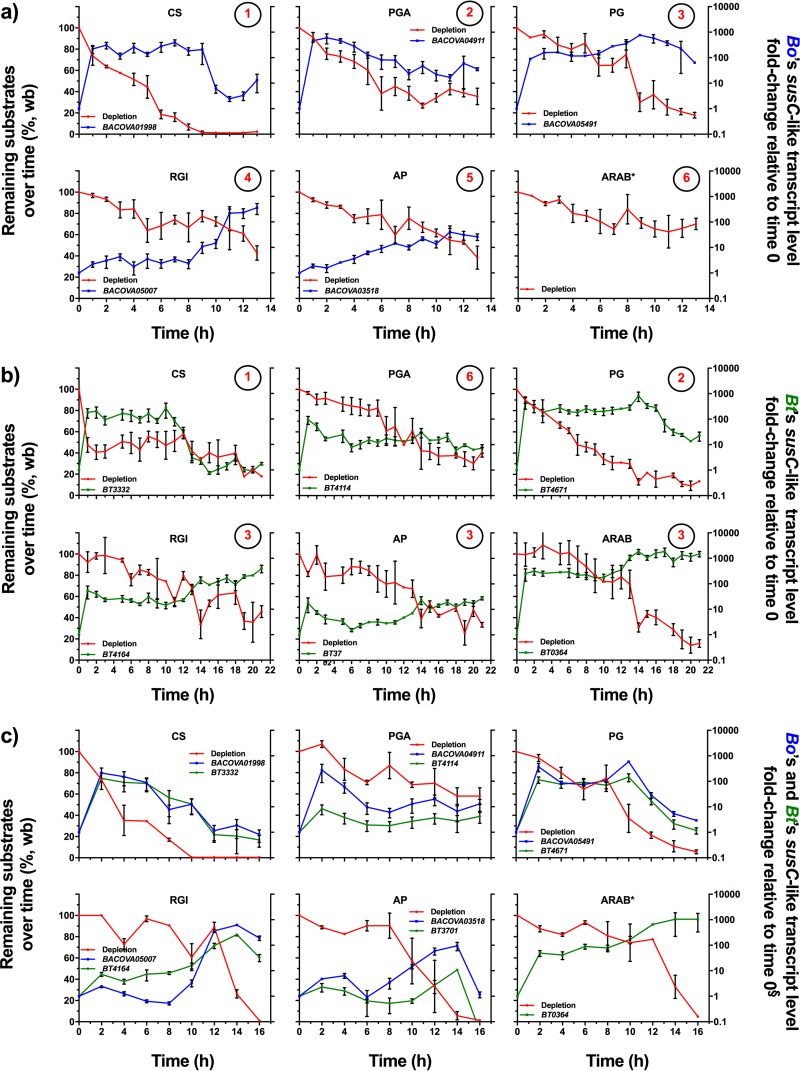
Individual glycan depletion in the mixtures and expression of PULs by bacteria species over time during growth. (a) Substrate depletion by *B. ovatus* (red lines) and temporal expression of PULs (blue lines). (b) Substrate depletion by *B. thetaiotaomicron* (red lines) and temporal expression of PULs (green lines). Circled numbers refer to the hierarchical ranking order, based on the following criteria: (i) the depletion slope of each glycan to the midpoint of the time course experiment and (ii) the final remaining level of each glycan. In case the two criteria were not in an agreement, the former was considered more important, since establishing the hierarchical preference ranking of the glycans was the main objective of the study. In panels a and b, rankings of RGI and AP could not be differentiated. (c) Substrate depletion by *B. ovatus* and *B. thetaiotaomicron* grown together (red lines) and temporal expression of *B. ovatus* PULs and that of *B. thetaiotaomicron* PULs (blue and green lines, respectively) (cocultured study). All analyses were performed using samples collected throughout the exponential growth phase of the corresponding bacteria (growth curves are given in Fig. S1a and b). Transcript levels were determined using qPCR against *susC*-like genes representing PULs which were triggered by each of the six glycans. Lines showing transcript level changes are described with locus tag numbers of corresponding genes. All transcript level changes were relative to time zero, prior to exposure to the mixture of six glycans. Error bars represent the standard errors of the means of three separate replicates. *, the arabinan utilization PUL of *B. ovatus* is not known; §, in the cocultured environment, *BT3701* gene expression (*susD*-like transcript level) was measured to determine the *B. thetaiotaomicron* responses to AP, rather than using the *BT3702* gene (*susC*-like transcript level), because of cross-reactivity to other genes in the qPCR assay (data not shown). No attempt was made to remeasure the *susD*-like transcript level of *B. thetaiotaomicron* in response to AP, since the expression levels of *susC* and *susD* during its growth on AP were not significantly different (31).

Direct measurement of carbohydrate utilization by colonic bacteria can be achieved by analyzing the disappearance of the individual glycans in a mixture. However, since many glycans share overlapping monosaccharide content, there must be a measurable unique structural feature for each glycan. In order to investigate direct glycan depletion in parallel with transcriptional activity, we chose a panel of six different glycans which could be differentiated based on at least one unique sugar or linkage present in each (Fig. 1). *In vitro* time course studies were conducted for *B. ovatus* and *B. thetaiotaomicron* grown either individually or together in a glycan mixture containing chondroitin sulfate (CS), polygalacturonic acid (PGA), PG, RGI, ARAB, and amylopectin (AP), each at an equal concentration so that the total carbohydrate concentration was 5 g/liter. In addition to harboring structural features that allowed their direct measurement in a mixture, these glycans were chosen because both bacteria are capable of degrading them (see Fig. S1a and b in the supplemental material). Medium samples and bacterial cells were collected at 1-h intervals during active growth in this mixture (Fig. S2c and d). At each time point, we measured the remaining glycans (Fig. 2, red lines) and transcription of sentinel genes contained in the corresponding PUL that targets each glycan (Fig. 2, blue lines for *B. ovatus* and green lines for *B. thetaiotaomicron*). To make sure there were no contaminating sugars produced by *B. ovatus* or *B. thetaiotaomicron* over time (e.g., in lipopolysaccharide or capsule), we performed parallel measurements in cultures grown on medium with mannose (Fig. S2), a sugar not contained in any of the glycans in our mixture, and we found that glucose was the only sugar detected during the active growth period (Fig. S2a and c) and was detected in only a negligible amount (Fig. S2b and d).

10.1128/mBio.01068-17.2 FIG S1  Growth curves of *B. ovatus* (a) and *B. thetaiotaomicron* (b) on individual glycans used in this study (5-mg/ml solutions). (c) *B. ovatus* growth on a mixture containing the six glycans. (d) *B. thetaiotaomicron* growth on the glycan mixture. The end of the exponential growth phase in each medium is shown with a dashed line. The error bars represent the standard errors of the means of three biological replicates. Download FIG S1, TIF file, 1.1 MB. Copyright © 2017 Tuncil et al. 2017 Tuncil et al. This content is distributed under the terms of the Creative Commons Attribution 4.0 International license.

10.1128/mBio.01068-17.3 FIG S2  Bacterial growth on mannose and the amount of bacterial glucose synthesis during growth. (a) *B. ovatus* growth on mannose; (b) amount of glucose synthesized by *B. ovatus* grown in this medium. (c) *B. thetaiotaomicron* growth on mannose; (d) amount of glucose synthesized by *B. thetaiotaomicron* grown in this medium. (e) Cell density of mannose-containing medium inoculated with both *B. ovatus* and *B. thetaiotaomicron* at an equal ratio (cocultured system); (f) amount of glucose synthesized. The end of the exponential growth phase in each medium is shown with a dashed line. Error bars represent the standard errors of the means for two separate replicates. Download FIG S2, TIF file, 1 MB. Copyright © 2017 Tuncil et al. 2017 Tuncil et al. This content is distributed under the terms of the Creative Commons Attribution 4.0 International license.

Based on direct glycan measurements, each species utilized the presented glycans in a hierarchical order, but with somewhat different priorities between species (Fig. 2a and b; circled numbers indicate hierarchy, as described in the figure legend). *B. ovatus* exhibited four different glycan depletion and PUL expression trends (Fig. 2a). The first one was rapid recognition (as evidenced by transcript activation) and immediate utilization, exemplified by CS and PGA. The second trend was observed in PG, in which transcript activation was very quick but glycan degradation started at a later time. The third was exemplified by RGI, where gene expression and utilization occurred at a later time that was after the most preferred glycan, CS, was gone. Lastly, for AP, a gradual decrease in its amount was seen as a gradual increase occurred in its gene expression. ARAB was hardly utilized by *B. ovatus*, consistent with a previous report (21); the corresponding PUL that enables weak growth is not known.

*B. thetaiotaomicron* showed three glycan recognition and utilization trends that were somewhat different from results with *B. ovatus* (Fig. 2b). The first trend, represented in cultures with CS and PG, showed recognition and utilization occurring rapidly after exposure to the mixture. The second trend was exemplified by PGA, where PUL transcription displayed a short-lived rise upon exposure to the glycan mixture, followed by a plateau state (Fig. 2b, green line). This was consistent with glycan depletion, where a small amount of PGA was utilized within the first hour, followed by no significant depletion until the mid-exponential phase (1 to 8 h), and then a significant decrease (*P* < 0.05, two-tailed Student’s *t* test) in the amount of remaining substrate (Fig. 2b, red lines). RGI, AP, and ARAB exemplified the third trend, where the PUL activation was initially rapid, followed by a plateau and then by a further increase. Depletion of these glycans occurred mostly after the second rise in PUL transcription, suggesting that *B. thetaiotaomicron* prepares its utilization systems for RGI, AP, and ARAB upon exposure to the glycan mixture but does not degrade them while utilizing the prioritized glycans CS and PG.

Using this approach of measuring carbohydrate utilization by glycan depletion, hierarchical rankings of glycans could be made, while they were less clear based on transcriptional findings. For example, in the case of *B. ovatus*, transcriptional profiling revealed only two types of preferences, immediate and delayed, while glycan depletion showed the four different utilization profiles described above. This may be attributed to the likelihood that features exist beyond transcription that influence glycan utilization trends, such as different efficiencies of the respective enzyme systems for digesting the glycans and variations in posttranslational processing and secretion of the synthesized proteins.

Consistent with previous reports, in which the presence of preferred polysaccharides was shown to repress *B. thetaiotaomicron*’s transcription for the utilization of lower-prioritized glycans (23–25), the presence of highly prioritized glycans appeared to repress transcription of those with lower priority, and this might explain how different bacteria set up their hierarchical preference for specific glycans. This can be seen in *B. ovatus*, where the PUL corresponding to RGI utilization was repressed until CS was fully utilized (Fig. 2a). Even though *B. thetaiotaomicron* showed an initial increase in transcription of all PULs, for the lower-prioritized glycans (RGI, AP, and PGA), this was followed either by a plateau state (e.g., RGI [no statistical difference was observed until 14 h; *P* < 0.05, two-tailed Student’s *t* test]) or a decrease in transcription (e.g., PGA and AP [statistically decreased at 6 h compared to 1 h; *P* < 0.05, two-tailed Student’s *t* test]), which could be interpreted as repression. Later in the experiment, the rapid decrease in CS transcription coincided with an increase in transcription of lower-prioritized glycans.

For some high-priority glycans, bacterial PUL transcription was surprisingly sensitive to even very low glycan concentrations, despite other low-priority glycans being more abundant (typified by *B. ovatus* CS utilization, PG utilization by both species, and *B. thetaiotaomicron* ARAB use). In other cases, bacterial sensitivities appeared to be more tightly tuned to the residual concentration of glycan in the medium (e.g., expression of PGA utilization genes by *B. ovatus*).

### Prioritization remains similar during coculture in a glycan mixture.

To test whether glycan metabolic hierarchies can change in a competitive environment, glycan depletion and PUL transcriptional profiles for corresponding glycans were compared when *B. ovatus* and *B. thetaiotaomicron* were grown singly versus together in the glycan mixture (Fig. 2c). While expression levels of PULs in both bacteria slightly varied in both magnitude and timing in coculture versus levels when cultured alone, overall expression patterns were similar to those of the singly cultured bacteria. This suggests that human gut symbionts are “programmed” to utilize glycans in a hierarchical order when they are presented together in a competitive environment.

### Reciprocal priorities for glycans may support community stability.

**FIG 3:**
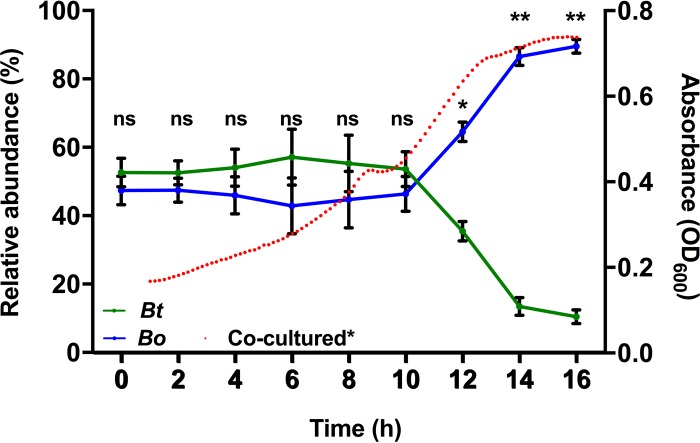
Relative abundances (the percentages of total bacteria) of *B. ovatus* (blue line) and *B. thetaiotaomicron* (green line) grown together in the mixture of six glycans, and the cell density of the medium, measured by absorbance (OD_600_) (red dots). Abundances were determined using qPCR with species-specific genes (*BACOVA03426* for *B. ovatus* and *BT3854* for *B. thetaiotaomicron*). Error bars represent the standard errors of the means of three separate replicates. Statistically significant differences were calculated using a two-tailed unpaired Student’s *t* test. *, *P* < 0.01; **, *P* < 0.0001; ns, not significant (*P* > 0.05).

The stability of the cocultured community was also monitored over the course of 16 h by determining the relative abundance of each bacterial species by using quantitative PCR (qPCR) using species-specific primers (Table S2). There was no significant change (*P* < 0.05, two-tailed Student’s *t* test) observed in the relative abundance of the two species in the first 10 h of incubation (Fig. 3), during which there was full or partial depletion of CS, PGA, PG, and AP (Fig. 2c, red lines). Afterwards *B. ovatus* dominated in growth during which RGI and ARAB were mainly utilized.

**FIG 4:**
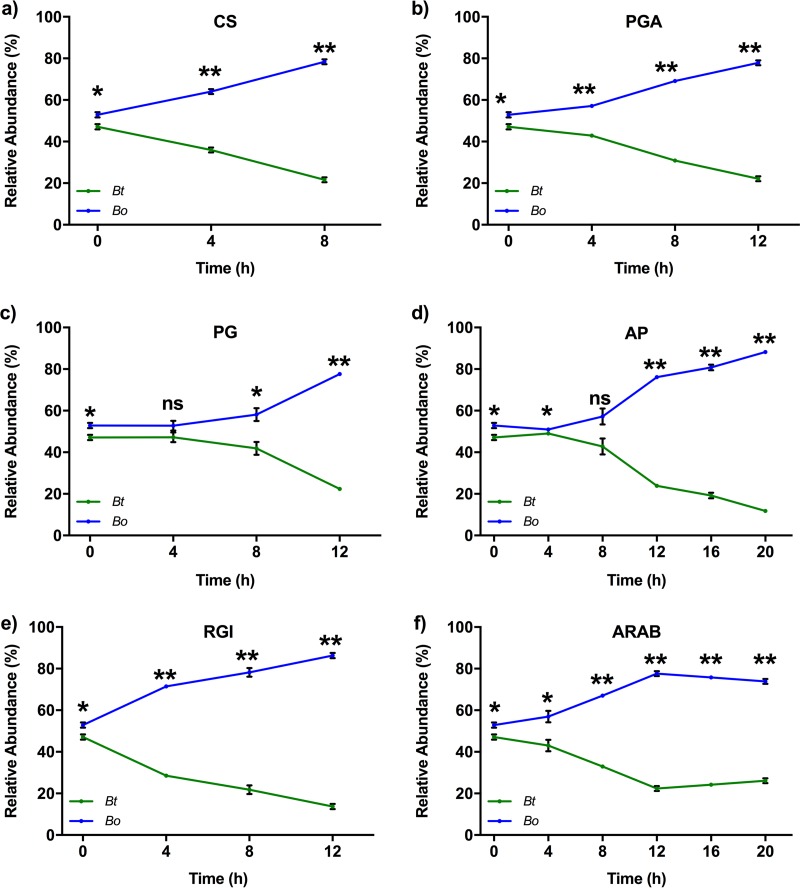
Relative abundances (percentages of total bacteria) of *B. ovatus* (blue line) and *B. thetaiotaomicron* (green line) grown together in media containing the individual glycans as the only carbon source. Abundances were determined using qPCR with species-specific genes (*BACOVA03426* for *B. ovatus* and *BT3854* for *B. thetaiotaomicron*). Error bars represent the standard errors of the means of three separate replicates. Statistically significant differences were calculated using a two-tailed unpaired Student’s *t* test. *, *P* < 0.05; **, *P* < 0.0001; ns, not significant (*P* > 0.05). Optical densities of the *B. ovatus*-*B. thetaiotaomicron* cocultures with these defined glycans are given in Fig. S3.

We wished to investigate what factors contributed to the stability of the community, rationalizing that it could either be (i) mutual utilization of the same sugars without one species dominating or (ii) in the time frame when one species was utilizing a particular glycan, the other was using a different glycan. The organisms were cocultured in a medium containing each of the individual glycans as the sole carbon source, and the relative abundance of each species was determined (Fig. 4). Although some substrates (PG and AP) promoted equal competition of the two species at early time points, in all cases *B. ovatus* outcompeted *B. thetaiotaomicron* for the individual glycans. This suggests that, in the first 10 h of incubation, *B. thetaiotaomicron* was able to persist because the two competing bacterial species utilized different glycans at different times so as not to compete directly with each other. This could be explained by the different hierarchical preferences that were exhibited by the bacteria for some of the same glycans. For example, *B. ovatus* utilized PGA as the second-ranked glycan, while *B. thetaiotaomicron* utilized it as its fourth-ranked glycan; similarly, *B. ovatus* utilized PG as its third-ranked glycan and *B. thetaiotaomicron* utilized it as its second-ranked glycan (Fig. 2a and b). Thus, it appears there could be reciprocal priorities for some glycans, which may allow the bacteria to maintain their coexistence in such a competitive environment.

10.1128/mBio.01068-17.4 FIG S3  Optical densities of the *B. ovatus*-*B. thetaiotaomicron* cocultures in media containing chondroitin sulfate (a), polygalacturonic acid (b), pectic galactan (c), rhamnogalacturonan I (d), amylopectin (e), and arabinan (f). Download FIG S3, TIF file, 0.6 MB. Copyright © 2017 Tuncil et al. 2017 Tuncil et al. This content is distributed under the terms of the Creative Commons Attribution 4.0 International license.

### Cooperation between species influences community dynamics.

During growth in the mixture of the six glycans, stability of the artificial community was disrupted after 10 h (Fig. 3), which paralleled a rapid depletion of RGI and ARAB (Fig. 2c), suggesting that both of these lower-priority glycans favored *B. ovatus*. In the case of RGI, two observations could explain why *B. ovatus* dominated, (i) *B. ovatus* RGI PUL expression increased suddenly after 10 h, indicating its ability at that point to rapidly utilize RGI (Fig. 2c), and (ii) the growth rate of *B. ovatus* on RGI (change of 0.032 optical density units at 600 nm per hour [OD_600_/h]) was more rapid than for *B. thetaiotaomicron* (0.022 OD_600_/h) (Fig. S1a and b).

**FIG 5:**
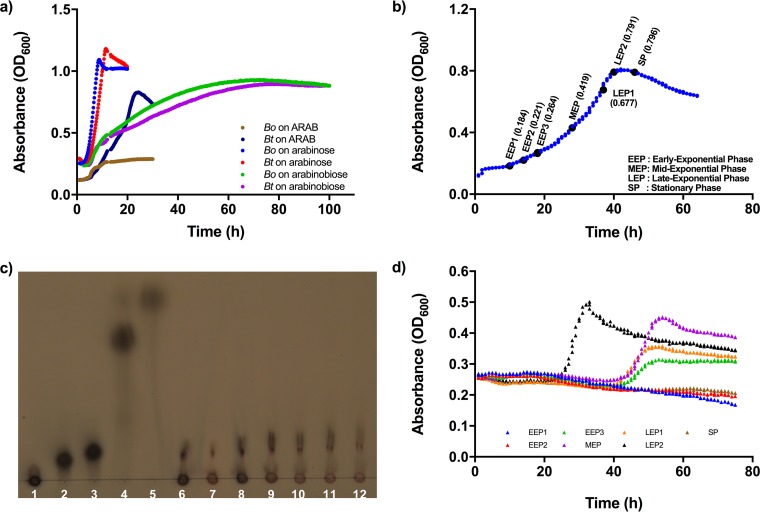
(a) Growth curves of *B. ovatus* and *B. thetaiotaomicron* grown individually in media containing different arabinan-based carbon sources. (b) Growth of *B. thetaiotaomicron* on medium containing ARAB as the only carbon source. Thicker black dots indicate points where cultures were harvested for subsequent analysis. Numbers in parentheses at each harvesting point refer to absorbance values. (c) Thin-layer chromatographic analysis of ARAB breakdown products in medium samples harvested throughout the growth phases of *B. thetaiotaomicron*. Columns 1 to 5 were external standards: 1, ARAB; 2, arabinoheptaose; 3, arabinohexaose; 4, arabinobiose; 5, arabinose. Columns 6 to 12 were samples: 6, EEP1; 7, EEP2; 8, EEP3; 9, MEP; 10, LEP1; 11, LEP2; 12, SP. (d) *B. ovatus* growth on medium samples harvested at different time points throughout *B. thetaiotaomicron*’s growth on ARAB. For instance, EEP1 refers to *B. ovatus* growth on medium harvested at the early exponential phase for *B. thetaiotaomicron*’s growth on ARAB.

More strikingly, for ARAB, since *B. ovatus* was unable to utilize it to a substantial degree (Fig. 2a; see also Fig. S1a), we hypothesized that cross-feeding of breakdown products allowed it to outcompete *B. thetaiotaomicron*. To test the ARAB hypothesis, we first monitored *B. ovatus* and *B. thetaiotaomicron* growth on arabinose, the building block of ARAB, and arabinobiose, an intermediate α-(1,5)-linked disaccharide proposed to be a product of ARAB degradation by *B. thetaiotaomicron*. Even though *B. ovatus* did not grow well on ARAB, it was able to utilize arabinose and arabinobiose as efficiently as *B. thetaiotaomicron* (Fig. 5a). *B. thetaiotaomicron* was then investigated for its ability to release ARAB breakdown products into the medium while growing on ARAB, which could then be utilized by *B. ovatus*. *B. thetaiotaomicron* was grown alone on a medium containing only ARAB, and samples were collected at different growth stages (3 samplings at early exponential phase [samples EEP1, EEP2, and EEP3], 1 sampling at mid-exponential phase [sample MEP], 2 samplings at late-exponential phase [LEP1 and LEP2], and 1 sampling at stationary phase [SP]) (Fig. 5b). The breakdown products observed in the culture supernatant were mainly arabinooligosaccharides with fewer than 7 arabinose units (Fig. 5c, lanes 6 to 12). *B. ovatus* did not grow on the media obtained at the EEP1 and EEP2 growth stages (Fig. 5d); we attributed this to a paucity of released ARAB breakdown products. However, it grew well on medium obtained after EEP2, with different degrees of growth culminating in a high growth rate in the LEP2 medium. Notably, the growth rate of *B. ovatus* on LEP2 (0.026 OD_600_/h) (Fig. 5d) was significantly faster (two-tailed Student’s *t* test, *P* < 0.0001) than that of *B. thetaiotaomicron* on ARAB (0.017 OD_600_/h) (Fig. 5b), demonstrating that *B. ovatus* more efficiently grows on ARAB breakdown products than *B. thetaiotaomicron* can on ARAB itself. Taken together, these findings suggest a reason for why *B. ovatus* outcompeted *B. thetaiotaomicron* in the later stages of coculture in which RGI and ARAB were the main resources competed for in the glycan mixture.

We also considered that *B. ovatus* might metabolize the capsular polysaccharides of *B. thetaiotaomicron*; however, this was not the case (see Text S1).

### Glycan length contributes to prioritization but is not the main factor in this phenomenon.

Because structural variations within a class of polysaccharides have been shown to drive different microbiota fermentation responses (30), we hypothesized that a change in a glycan structure may alter its ranking in the hierarchy. To test this, the hierarchical glycan utilization experiment was repeated with *B. ovatus* with replacement of one of the glycans, AP, to a simpler AP molecular structure [maltohexaose (MH), a degree of polymerization (DP) 6 α-(1,4)-glucose-linked chain, which is representative of the longer linear chains found in AP]. The rationale for the selection of MH as a substitute was based on a preliminary experiment in which 13 different normal and waxy starches, and starch oligomer analogs, bearing different structural features, were tested for their effects on growth of *B. ovatus* (Fig. S5; Table S1). *B. ovatus* utilized lower-molecular-size starches faster than higher-molecular-size ones. Thus, MH was chosen because it has a DP that is at least 7 to 8 orders of magnitude shorter than the AP from maize but still requires the *Bacteroides* SUS for processing (18, 31).

10.1128/mBio.01068-17.5 FIG S4  Relative abundances (percentages of total bacteria) of (a) wild-type (WT) *B. ovatus* (blue line) and WT *B. thetaiotaomicron* (green line) and (b) WT *B. ovatus* (blue line) and acapsular (ΔCPS) *B. thetaiotaomicron* (green line) grown together in medium containing arabinan as the sole carbohydrate source. (c) Comparison of relative abundances of WT *B. thetaiotaomicron* and ΔCPS *B. thetaiotaomicron*, which were cocultured with WT *B. ovatus*, at time zero and 20 h after incubation. Abundances were determined using qPCR targeting species-specific genes (*BACOVA03426* for *B. ovatus*; *BT3854* for *B. thetaiotaomicron*). Error bars represent the standard errors of the means of three separate replicates. Statistically significant differences were calculated using a two-tailed unpaired Student’s *t* test. *, *P* < 0.0001; **, *P* < 0.01; ***, *P* < 0.05; ns, not significant (*P* > 0.05)]. Download FIG S4, TIF file, 0.8 MB. Copyright © 2017 Tuncil et al. 2017 Tuncil et al. This content is distributed under the terms of the Creative Commons Attribution 4.0 International license.

10.1128/mBio.01068-17.6 FIG S5  Growth curves of *B. ovatus* grown on different starches. Download FIG S5, TIF file, 1.5 MB. Copyright © 2017 Tuncil et al. 2017 Tuncil et al. This content is distributed under the terms of the Creative Commons Attribution 4.0 International license.

10.1128/mBio.01068-17.9 TABLE S1  Structural features of starches used and growth parameters of *B. ovatus* on these starches. Download TABLE S1, DOCX file, 0.3 MB. Copyright © 2017 Tuncil et al. 2017 Tuncil et al. This content is distributed under the terms of the Creative Commons Attribution 4.0 International license.

**FIG 6:**
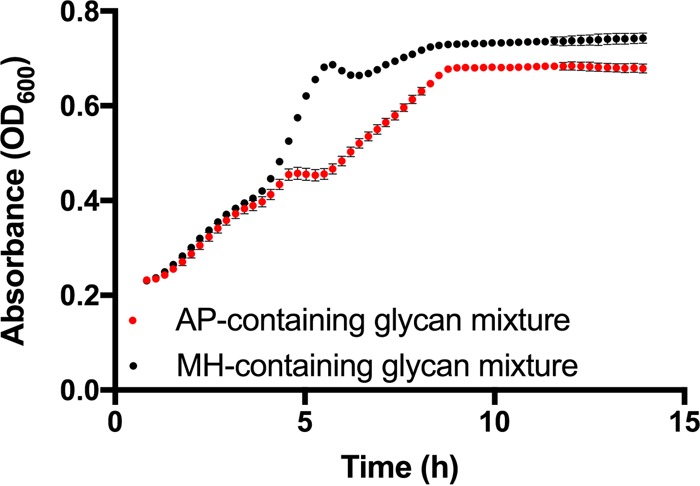
Growth curves of *B. ovatus* grown on the AP-containing glycan mixture (red dots) and MH-containing glycan mixture (black dots). Error bars represent the standard errors of the means of three biological replicates.

Consistent with our hypothesis, *B. ovatus* showed different growth patterns on AP- and MH-containing glycan mixtures, with differences observed in the second half of the growth period (Fig. 6), suggesting that ranking orders of highly preferred glycans were not altered but a change in the hierarchical preference order of less-preferred glycans occurred.

**FIG 7:**
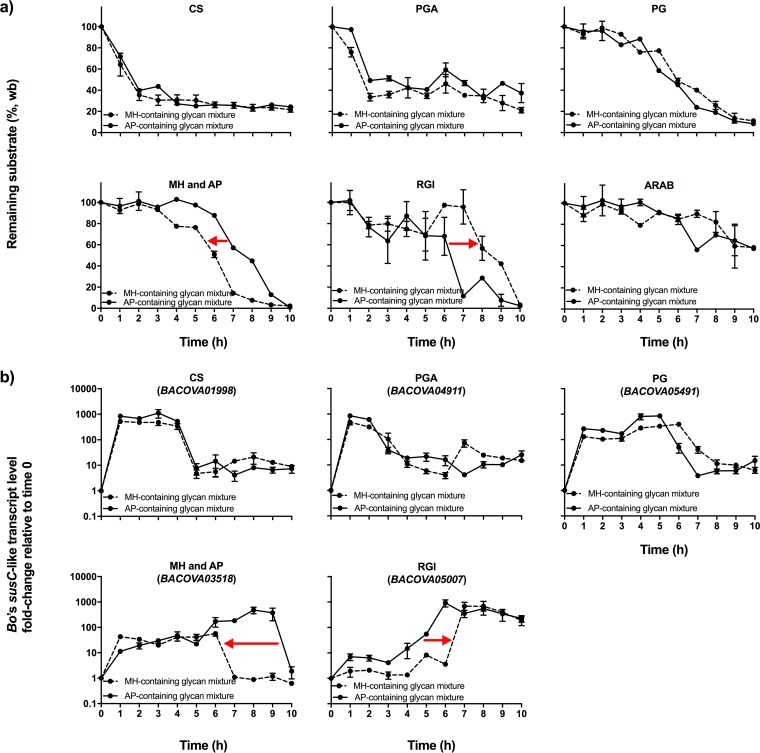
Glycan utilization and corresponding PUL expression by *B. ovatus* grown on an AP-containing glycan mixture (solid lines) and on an MH-containing glycan mixture (dashed lines). (a) Substrate depletion; (b) temporal expressions of PULs. All analyses were performed using samples collected throughout exponential growth phase of each species (Fig. 6). Lines showing transcript level changes are described with locus tag numbers of corresponding genes. All transcript changes are shown relative to time zero. Error bars represent the standard errors of the means of three separate replicates. *, the ARAB utilization PUL of *B. ovatus* is not known.

Hierarchical preference order changed, with MH moving one ranking above AP, with a coinciding delay in RGI degradation in the MH-containing glycan mixture (Fig. 7a). Degradation of MH began at 5 h, compared to AP at 7 h, which caused a delay in RG1 degradation from 6 to 7 h. The PUL for starch (*BACOVA03518*) returned to basal expression 3 h earlier when MH was substituted for AP, coinciding with the earlier depletion of MH (Fig. 7b). Conversely, expression of the RGI-targeting gene (*BACOVA05007*) was delayed when MH was present. For AP, a gradual increase started at 3 h after incubation and reached its maximum level at 6 h after incubation; for MH, the sudden increase in its expression was between 6 and 7 h. The preferred glycans CS, PGA, and PG showed unaltered hierarchical preference, and PULs targeting these glycans showed similar expression and repression patterns in both glycan mixtures. Thus, glycan structure is an important factor in determining the hierarchical preference for *B. ovatus*, but there are clearly additional repression signals that cannot be overcome by even an extreme change in glycan size.

### *B. ovatus* preferentially utilizes amylose over amylopectin.

**FIG 8:**
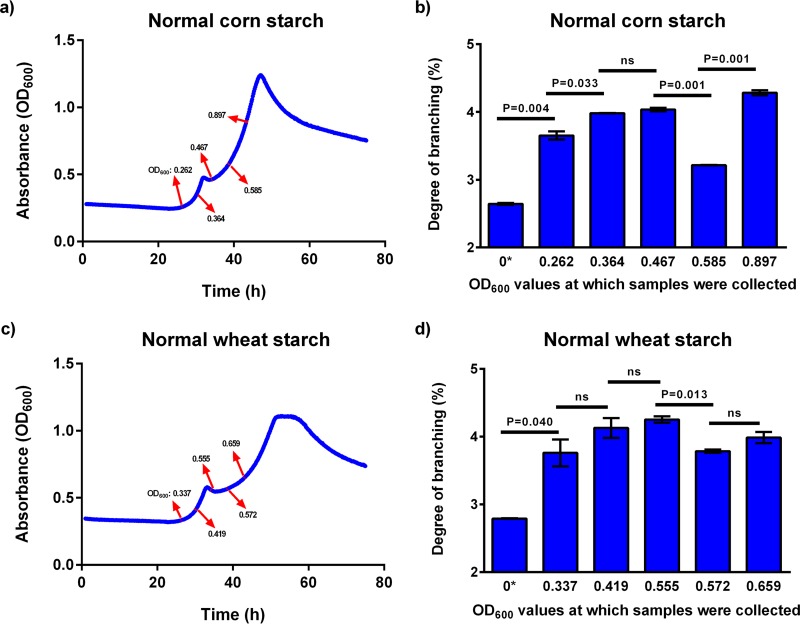
(a and b) Growth curves of *B. ovatus* on normal corn starch (a) and corn starch with changes in the degree of branching (b). (c and d) Growth curves of *B. ovatus* on normal wheat starch (c) and wheat starch with changes in the degree of branching (d). Red arrows represent the points at which medium samples were collected for determination of the degree of branching using ^1^H NMR. Error bars represent the standard errors of the means of two separate replicates. Statistically significant differences were calculated using a two-tailed unpaired Student’s *t* test. ns, not significant (*P* > 0.05).

Within starch, there are two principal molecules, amylose and amylopectin, and *B. ovatus* was found to first prefer the simpler amylose structure (linear) and then the more complex amylopectin structure (highly branched). *B. ovatus* was found to exhibit diauxic growth on normal starches but not on waxy starches, which contain only amylopectin (Fig. S5). Initial growth rates (until the pause points) were also faster on normal starches and were positively correlated with amylose content (*r* = 0.842, *P* = 0.017). Therefore, we hypothesized that *B. ovatus* prefers the simpler linear amylose structure. To test this hypothesis, *B. ovatus* was grown on normal corn and wheat starches; medium samples were collected at different time points (Fig. 8a and c) and changes in the degree of branching of starches were monitored using ^1^H nuclear magnetic resonance (NMR). Prioritization of amylose over amylopectin would increase the degree of branching. Indeed, after early growth had initiated, there were large increases in degree of branching, supporting the idea that *B. ovatus* catabolized amylose with preference over amylopectin (Fig. 8b and d). After the exponential growth phase resumed, there were significant decreases in the degree of branching of the starches (between OD_600_ of 0.467 and 0.585 for normal corn starch [Fig. 8b] and between OD_600_ of 0.555 and 0.572 for normal wheat starch [Fig. 8d]), suggesting that amylopectin in both cases was utilized by *B. ovatus* after the diauxic shift. During this second growth phase, *B. ovatus* apparently first attacked branch points [α-(1,6)-linkages] of amylopectin, perhaps as well as linear chains, leading to a transient decrease in branches before degrading newly released linear chains.

Many bacteria have the ability to synthesize glycogen (32), which bears both α-(1,4) and α-(1,6) glucose linkages, as does starch, and they also express capsular polysaccharides (33), which could contain one or both of these linkages. Thus, we also considered that *B. ovatus* might produce glycogen or capsular polysaccharides that could interfere with the NMR analysis for degree of branching. However, this was not the case (see Text S1).

10.1128/mBio.01068-17.1 TEXT S1  Additional results and discussion, along with descriptions of other experimental procedures used. Download TEXT S1, DOCX file, 0.03 MB. Copyright © 2017 Tuncil et al. 2017 Tuncil et al. This content is distributed under the terms of the Creative Commons Attribution 4.0 International license.

## DISCUSSION

Determining the glycan utilization strategies of the members of the gut microbiota has become an important research focus due to the essential role of these dietary compounds on community structure, which is associated with several aspects of human health. To date, such studies (20, 22–25) have relied solely on transcriptional profiling of bacteria during growth on individual glycans, which does not necessarily reflect their actual utilization in more complex mixtures. Here, using carbohydrate depletion analysis in conjunction with transcriptional analysis, we investigated how *B. ovatus* and *B. thetaiotaomicron* utilize a complex glycan mixture, which might be more similar to the complex *in vivo* environment. Transcriptional profiling was not always an accurate measure of glycan utilization patterns. For example, even though there was a more-than-100-fold increase in transcription of the *B. thetaiotaomicron* ARAB-corresponding PUL, the ARAB substrate was not utilized until the mid-exponential growth phase (Fig. 2b). A similar trend was observed for *B. ovatus* PG utilization (Fig. 2a). This may have been due to posttranscriptional events, such as translation, trafficking to limited space on the membrane surface, or enzyme kinetics (34, 35). Also, the synthesized carbohydrate-degrading enzymes may not be optimal for the given glycan structures; hence, a high transcriptional rate could pair with slow utilization. Optimal alignment of carbohydrate structures to gut bacterial utilization systems is likely a factor in this phenomenon (8).

Our results show for the first time that hierarchical preferences of bacteria to dietary glycans are preserved when they are in a competitive environment, suggesting that bacteria are “programmed” to utilize different glycans in a specific order. In agreement with the findings reported by Pudlo et al. (25), different *Bacteroides* spp. were found to exhibit different hierarchical orders for the same glycan mixture. Further, our data suggest that for bacteria utilizing the same set of dietary glycans, reciprocal processing allows them to access glycan substrates simultaneously and survive in the competitive environment of the colon.

*B. thetaiotaomicron* and *B. ovatus* exhibited variable sensitivities to the different glycans, whereby gene recognition of certain glycans was concentration dependent (e.g., PGA in Fig. 2a), while in others, transcription levels remained high even with low residual glycan amounts (e.g., CS in Fig. 2a). A clear trend appeared where highly prioritized glycans were utilized while genes involved in utilization of lower priority glycans experienced at least some associated gene repression. This would be a reasonable way that bacteria would be able to establish glycan hierarchies whereby they could compete well on mixtures of dietary glycans. This might also be conceptually important in developing approaches to promote specific bacteria or bacterial groups by dietary supplementation, in that one would need to make sure that a selected glycan would be delivered to the target bacteria in an appropriate amount.

Even though MH was easily hydrolyzed (revealing a high growth rate compared to the other glycans [Table S1; Fig. S5]), substituting AP with MH only increased its rank by one place in the hierarchical order and did not change the rank order of the highly prioritized glycans (Fig. 7). This clearly indicated that glycan length contributes to prioritization, but there are other important factors to hierarchical placement of glycans (e.g., linkage type, monosaccharide composition). The increase in prioritization for MH versus AP went along with a delay in RGI degradation. More strikingly, replacing AP with MH repressed the expression of the *B. ovatus* RGI utilization gene at early time points. Within the first hour of incubation on AP, a 7-fold increase was observed in the *B. ovatus* RGI utilization gene, whereas there was only a 1.9-fold increase in this gene in the MH-containing mixture. In the MH-containing mixture, *B. ovatus* achieved maximum expression levels for the RGI utilization gene later than in the AP-containing mixture. Thus, glycan chemical structure can affect not only hierarchical placement but also transcriptional profiles.

Structures within a carbohydrate class were also found to be an important factor affecting the bacterial response to a given glycan. The linear starch amylose was utilized prior to amylopectin (Fig. 8). Better enzyme activities of *B. ovatus* starch-degrading enzymes on the straight-chain amylose might be the reason, as branch points in starch amylopectin are known to be harder to digest (36). A second possibility is that *B. ovatus* starch-binding proteins may more strongly bind amylose than amylopectin, thus causing *B. ovatus* to prioritize amylose. In support of this, the SusD-binding protein of *B. thetaiotaomicron* had higher affinity toward cyclic maltooligosaccharides that mimic the helical structure of amylose (31). A previous study revealed that glycan prioritization by bacterial species occurs because the monosaccharide units of the most-preferred glycans repress transcription of the genes responsible for catabolism of the least-preferred glycans. In one case, this required a small intergenic region in a transcript-autonomous region (25). However, for starch there is only one monosaccharide unit (glucose). Thus, our findings show that structural aspects of glycans affect prioritization, including linkage profiles, conformation, and molecular size.

Additionally, and in agreement with previous reports (37–41), cross-feeding of polysaccharides between bacterial species was shown to be an important determinant of microbial community structure. Coculturing of *B. thetaiotaomicron* and *B. ovatus* enabled *B. ovatus* to utilize previously inaccessible ARAB due to release of ARAB breakdown products (Fig. 5a and d). This indicated that *B. thetaiotaomicron* initiates ARAB degradation, which results in release of ARAB breakdown products into its growing environment. This further suggests some microbial species are programmed to feed neighboring counterparts.

*Bacteroides* species have been reported to produce SCFAs of acetate, propionate, and succinate (40, 42), and we also considered whether these produced SCFAs may contribute to community structure through cross-feeding. An experiment was designed to test whether these organisms could utilize SCFAs, which could explain the enhanced growth of one in the presence of another strain. Each strain was grown on 10 mM (final concentration for each) acetate, propionate, and butyrate, but no growth was observed (data not shown). Thus, cross-feeding was likely due to sugars, not SCFAs.

While our study and most previous studies have been conducted with purified and extracted, soluble fibers (similar to prebiotics), the additional barriers to utilization entailed with more natural, insoluble, and complex fiber presentations (i.e., in whole grains and vegetables) need to be explored to fully understand the relationship between diet and microbiota responses. However, the current type of study is necessary to provide an essential foundation for those more complex studies.

In conclusion, using the benchmark carbohydrate depletion method for assessing carbohydrate utilization by bacteria, in conjunction with PUL transcriptional analysis, a number of new insights were gained into dietary glycan utilization strategies of human gut bacteria. Glycan hierarchical preferences were established in two common gut *Bacteroides* species, *B. ovatus* and *B. thetaiotaomicron*, which exhibit partially reciprocal priorities to glycans. Highly prioritized glycans repressed gene expression of lower-prioritized ones, which might be the way that bacteria set up glycan hierarchies. The bacteria adhered to their hierarchical preference order even in a competitive environment, which may allow them to maintain their coexistence in a competitive environment. Finally, we found that structural features of glycans determine their place in the hierarchy and that bacteria differentiate utilization of carbohydrates within a glycan class. This study provides new information about strategies of bacterial species to utilize different dietary fibers when they are present as a mixture.

## MATERIALS AND METHODS

### Glycans used.

Arabinan came from sugar beet (product code [PC] P-ARAB), pectic galactan was obtained from potato (PC P-PGAPT), polygalacturonic acid was from citrus pectin (PC P-PGACT), and rhamnogalacturonan I was from potato (PC P-RHAM1); these glycans were all purchased from Megazyme International (Wicklow, Ireland). Amylopectin from maize (PC 10120) and chondroitin sulfate from bovine trachea (PC C9819) were purchased from Sigma-Aldrich (St. Louis, MO). The chemical structures of the glycans are illustrated in Fig. 1. Stocks for each glycan (10 mg/ml) were prepared using purified water and sterilized by autoclaving for 20 min at 121°C.

### Bacterial strains and growth on pure glycans.

*B. ovatus* ATCC 8483 and *B. thetaiotaomicron* VPI 5482 (ATCC 29148) strains were used for all experiments. The ΔCPS *B. thetaiotaomicron* strain was previously constructed by Rogers et al. (24).

*B. ovatus* and *B. thetaiotaomicron* were pregrown in chopped meat broth overnight at 37°C in an anaerobic chamber (Coy Laboratory Products Inc., Grass Lake, MI) under an 85% N_2_, 5% CO_2_, and 10% H_2_ atmosphere. Growth profiles of bacterial strains on pure glycans were determined anaerobically using a custom carbohydrate array constructed in a 96-well format as previously described (21). Briefly, 100 μl of each sterilized glycan stock (10 mg/ml) was loaded into each well of a 96-well plate. One-milliliter aliquots of bacterial cultures were centrifuged to pellet the cells, which were then washed with 1 ml of minimal medium (MM) containing no carbon source (MM was prepared as described in reference 20). These washed cells were used to inoculate 50 ml of 2× MM containing no carbon source. Resuspended cells (100 μl) were inoculated into each well containing 100 μl of glycan to obtain 200-μl cultures (5 mg/ml, final glycan concentration). Plates were sealed in an anaerobic chamber under the atmospheric conditions given above. Absorbance was measured at 600 nm (OD_600_) at 10- to 15-min intervals for 96 h by using a Powerwave HT absorbance reader coupled with a BioStack automated plate handling device (Biotek Instruments, Winooski, VT). Each species was tested in six replicates for each glycan. Data processing was handled using Gen5 software (BioTek) and GraphPad Prism version 7 software (GraphPad Software, Inc., La Jolla, CA).

### Glycan mixture exposure, growth in glycan mixtures, and sample collection.

In order to prepare *B. ovatus* and *B. thetaiotaomicron* for glycan mixture exposure, cells pregrown in chopped meet medium were individually pregrown to mid-exponential phase (OD_600_, 0.5 to 0.8) in MM containing glucose. The cells were then pelleted and washed with MM containing no carbon source prior to exposure to glycan mixtures (24). Bacterial cells were inoculated in triplicate into an equal volume of the glycan mixture (the final total glycan concentration was 5 mg/ml, and the final volume for each replicate was 120 ml) in an anaerobic chamber under the atmospheric conditions specified above. For singly cultured experiments (the experiments shown in Fig. 2a and b), two aliquots were removed every hour throughout their exponential growth phase (Fig. S1c and d): one for measuring the remaining glycans in the medium (4 ml) and the other for monitoring PUL expression over time relative to time zero (1 ml) (see Text S1 for a description of sample collection for the experiment where the effects of glycan structure on its prioritization by *B. ovatus* were studied [the experiments shown in Fig. 7]). For the coculture experiment, equal amounts of *B. ovatus* and *B. thetaiotaomicron* were inoculated into the same glycan mixture, and three aliquots were collected every 2 h in the exponential phase (Fig. 3, red dots): one for measuring the remaining glycans in the medium (4 ml), one for monitoring PUL expression over time relative to time zero (1 ml), and the last used to determine the relative abundance of each bacterial species over time (1 ml).

### Measurement of residual glycans in culture media.

Collected samples for glycan analysis were dialyzed (cutoff, 1 kDa; Spectrum Laboratories, Rancho Dominguez, CA) against purified water for at least 36 h to remove vitamins and minerals from the minimal medium as well as compounds possibly produced by bacterial species during growth on the glycan mixture, followed by lyophilization. Neutral and acidic monosaccharides found in the samples were determined on a weight basis by using gas chromatography as their trimethylsilyl (TMS) derivatives (43). Glycosyl linkage compositions of the samples were determined using gas chromatography coupled with mass spectrometry (GC/MS; 7890A and 5975C inert MSD with a triple-axis detector; Agilent Technologies, Santa Clara, CA) as their partially methylated alditol acetate derivatives (44). Neutral monosaccharide amounts were confirmed as their alditol acetate derivatives using GC/MS as described previously, which also allowed us to make sure the methylation steps performed during glycosyl linkage composition analysis were successful (44). For the experiment shown Fig. 7, different procedures were used for measuring the remaining glycans (see Text S1 in the supplemental material for details).

Over the time course experiment, the remaining AP, CS, RGI, and PG percentages relative to their initial amounts were determined by measuring glucose, glucuronic acid, rhamnose, and galactose amounts in the samples, respectively. Similarly, the remaining ARAB amount was measured by quantifying the remaining 5-arabinose linkage over time. The remaining PGA amount was calculated by subtracting the amount of total rhamnose from the amount of total galacturonic acid. These monosaccharides or linkages were chosen to determine the remaining corresponding glycan because they are unique signatures of each glycan found in the mixture (Fig. 1).

Members of the *Bacteroides* genus possess the ability to synthesize capsular polysaccharides (33, 45–47). To make sure that the bacterial species tested in this study did not synthesize any glycan that could interfere with our glycan analysis, in parallel with our time course assays other time course studies were done in which these bacterial species were grown in medium containing mannose as the only carbon source; these samples were collected for glycan analysis at each time point during their exponential growth phase (Fig. S2a, c, and e). Glucose was the only monosaccharide detected in these media, but its amounts were negligible (Fig. S2b, d, and f).

### Transcriptional analysis of PUL genes by qPCR.

PUL expression analyses were performed as previously described (24). Briefly, cells were harvested by centrifugation of the aliquots at 13,000 rpm for 10 min; cells were then treated with RNAprotect (Qiagen) and stored at −80°C until further processing. Total RNA was extracted from cells using an RNeasy minikit (Qiagen) and treated with Turbo DNase I (Ambion). Reverse transcription was performed using SuperScript III reverse transcriptase (Invitrogen) according to the manufacturer’s instructions. cDNA quantification was performed with a Mastercycler EP Realplex system (Eppendorf) using SYBR Fast qPCR master mix (Kapa Biosystems, Inc., Wilmington, MA) for 40 cycles of 95°C for 3 s, 55°C for 20 s, and 72°C for 8 s. All transcript levels were normalized based on 16S rRNA abundance, and transcript levels at time zero were used as references. Primers used are given in Table S2; they targeted previously validated sentinel *susC*-like genes, as previously reported (21).

10.1128/mBio.01068-17.10 TABLE S2  Primers used in this study. Download TABLE S2, DOCX file, 0.1 MB. Copyright © 2017 Tuncil et al. 2017 Tuncil et al. This content is distributed under the terms of the Creative Commons Attribution 4.0 International license.

### qPCR enumeration of competing species in coculturing experiments.

Cells were harvested by centrifugation for quantification. DNA was isolated using a DNeasy blood and tissue kit according to the manufacturer’s instructions (Qiagen). DNA (10 ng) was assayed in duplicate in a Mastercycler EP Realplex system (Eppendorf) using SYBR Fast qPCR master mix (Kapa Biosystems, Inc., Wilmington, MA) and species-specific primers for 40 cycles of 95°C for 3 s, 55°C for 20 s, and 72°C for 8 s. Species-specific primers used for *B. ovatus* and *B. thetaiotaomicron* were *BACOVA03426* and *BT3854*, respectively (Table S2). Purified genomic DNA standards (range, 80, 20, 2, 0.4, 0.08, and 0.01 ng) of each species were included in duplicate in each qPCR run. A standard curve generated from these standards was used to calculate the relative abundances of species in each sample.

See Text S1 for descriptions of other experimental procedures used in our study.

10.1128/mBio.01068-17.7 FIG S6  Carbohydrates synthesized by *B. ovatus* while growing on glucose and native wheat starch. (a) *B. ovatus* growth on glucose. Thicker red dots indicate the points where cultures were harvested for subsequent analysis. (b) Glucose amounts detected in the medium samples harvested throughout *B. ovatus* growth on glucose (red dots in panel a). (c) Monosaccharides (other than glucose) detected in the medium harvested throughout *B. ovatus* growth on glucose (red dots in panel a). (d) *B. ovatus* growth on normal wheat starch. Thicker red dots indicate the points where cultures were harvested for subsequent analysis. (e) Glucose amounts detected in the medium samples harvested throughout *B. ovatus* growth on normal wheat starch (red dots in panel d). (f) Monosaccharides (other than glucose) detected in the medium samples harvested throughout *B. ovatus* growth on normal wheat starch (red dots in panel d). Error bars represent the standard errors of the means of two separate replicates. Download FIG S6, TIF file, 1.4 MB. Copyright © 2017 Tuncil et al. 2017 Tuncil et al. This content is distributed under the terms of the Creative Commons Attribution 4.0 International license.

10.1128/mBio.01068-17.8 FIG S7  (a) Absorbance changes of filtered medium samples harvested throughout *B. thetaiotaomicron*’s growth on ARAB over time (the negative control for Fig. 5d). Thick black dots in Fig. 5b indicate the points where the medium samples were harvested. For example, EEP1 refers to the absorbance change of the medium harvested at the early exponential phase of *B. thetaiotaomicron*’s growth on ARAB. (b) Growth of *B. thetaiotaomicron* on medium containing arabinose as the only carbon source. Thick black dots indicate the points where the medium was harvested for monitoring the growth of *B. ovatus*. The numbers in parentheses next to each harvested point refer to the absorbance values. (c) *B. ovatus* growth on medium harvested at different time points throughout *B. thetaiotaomicron*’s growth on arabinose. For example, LP1 refers to *B. ovatus* growth on medium harvested at the lag phase of *B. thetaiotaomicron*’s growth on arabinose. (The data in panels b and c represent results for the positive controls for Fig. 5b and d.) (d) Absorbance changes of the medium samples harvested throughout *B. thetaiotaomicron*’s growth on arabinose over time (these results represent the negative control for panel c). Download FIG S7, TIF file, 1.4 MB. Copyright © 2017 Tuncil et al. 2017 Tuncil et al. This content is distributed under the terms of the Creative Commons Attribution 4.0 International license.
