# Supplementary figures and images for: Reciprocal Prioritization to Dietary Glycans by Gut Bacteria in a Competitive Environment Promotes Stable Coexistence
Source: mBio. 2017 Oct 10;8(5):e01068-17. doi: 10.1128/mBio.01068-17 (PMC5635687; doi:10.1128/mBio.01068-17)

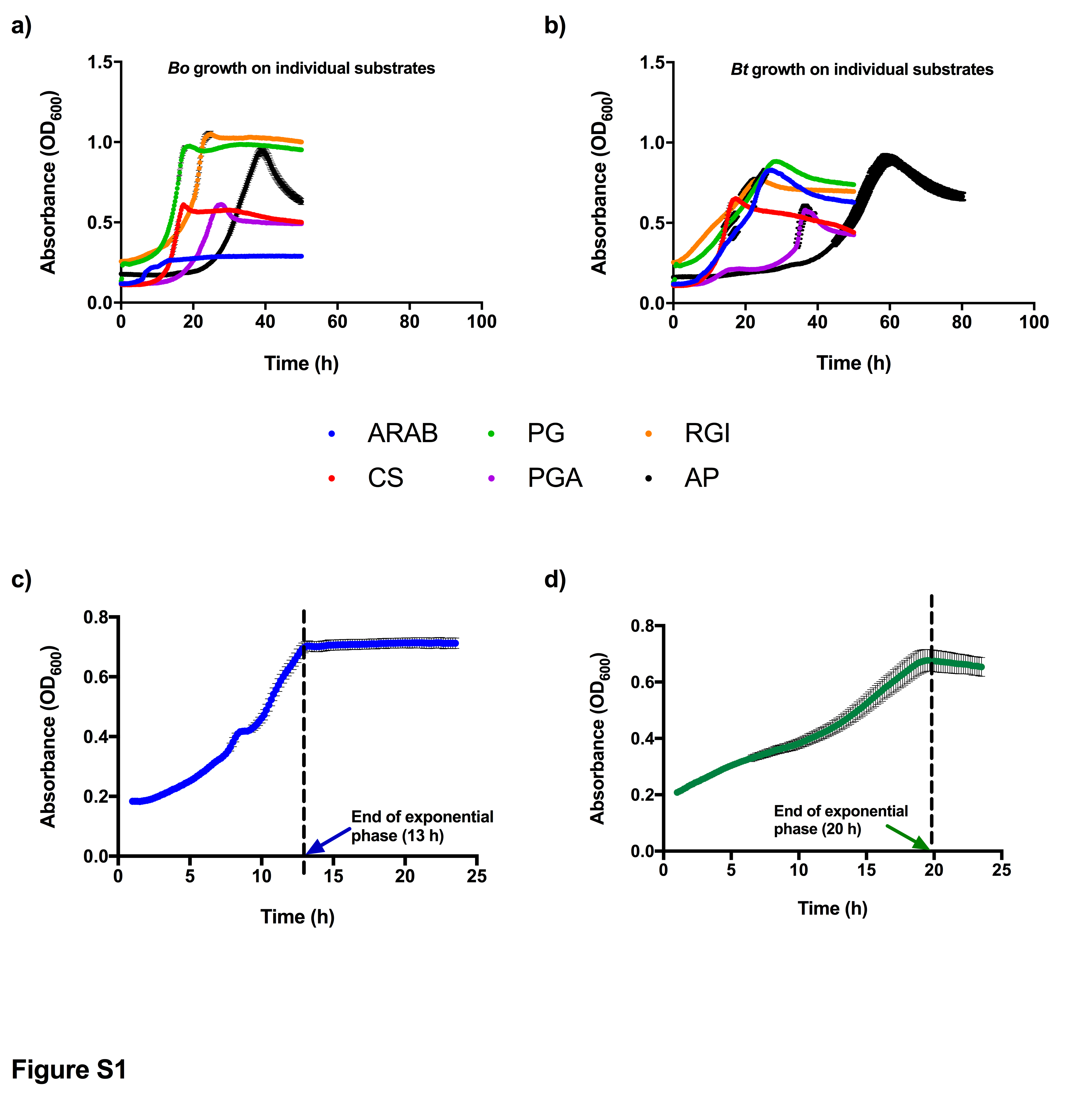

Supplement: FIG S1 [file mbo005173504sf1.tif]

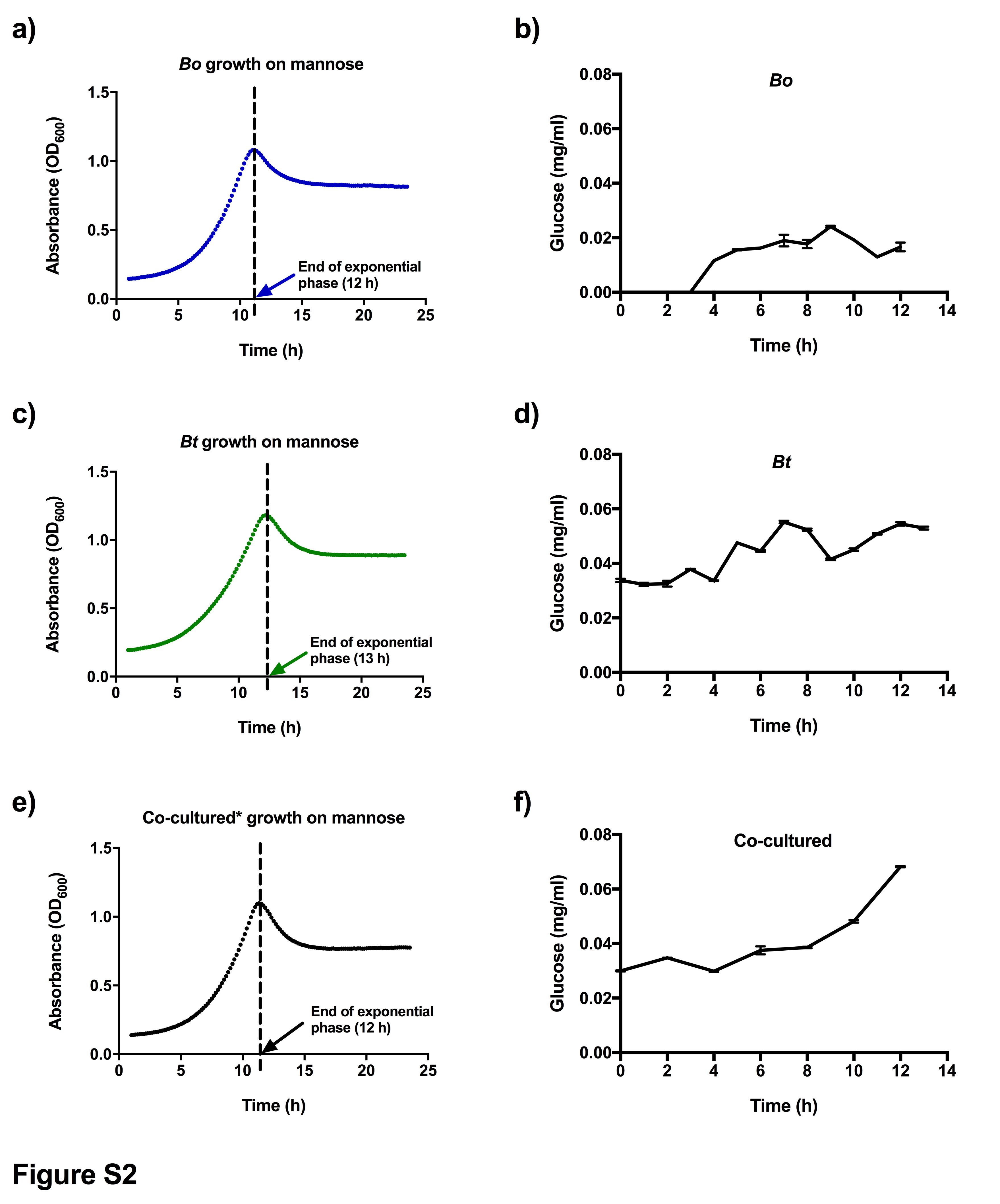

Supplement: FIG S2 [file mbo005173504sf2.tif]

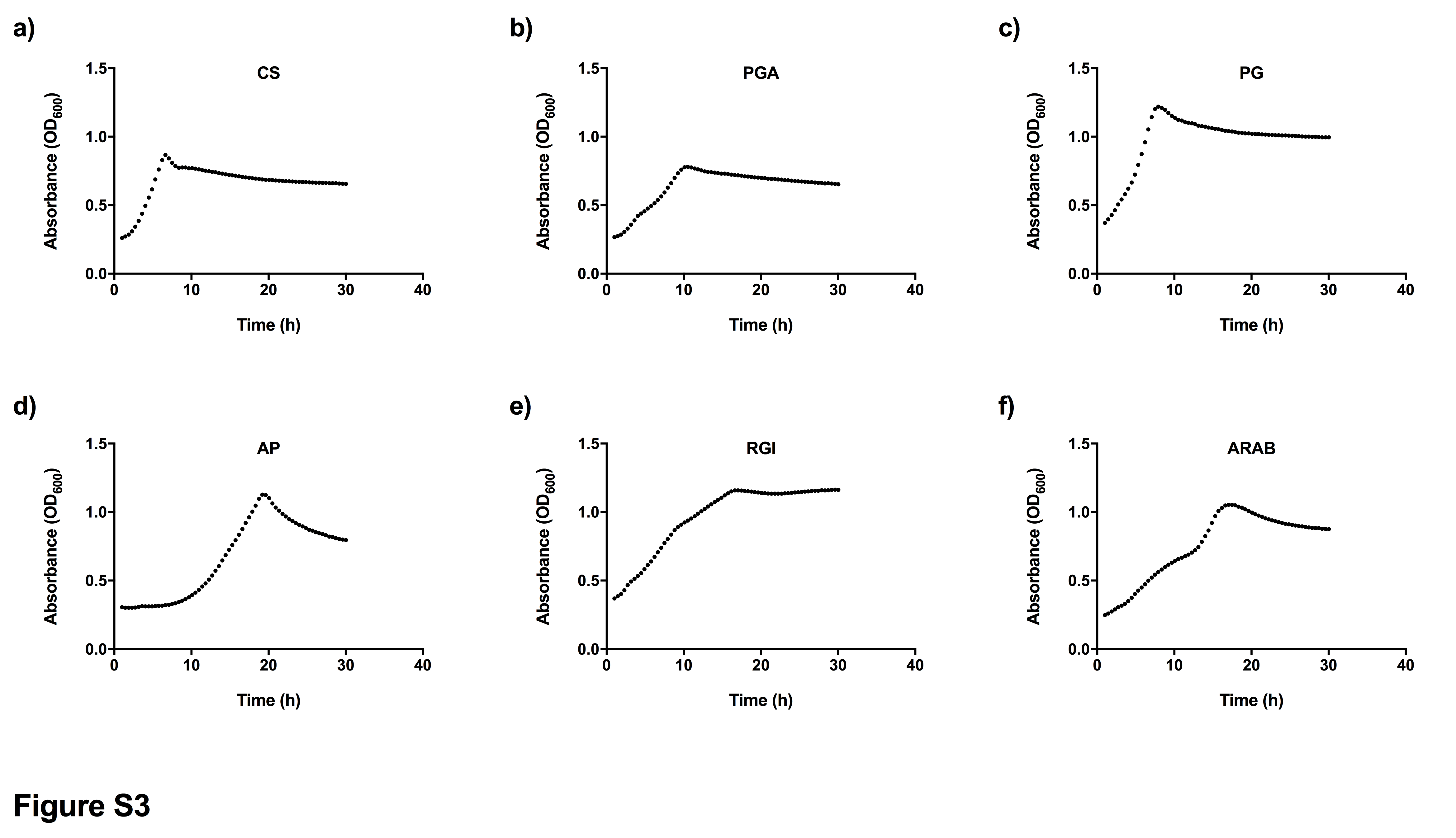

Supplement: FIG S3 [file mbo005173504sf3.tif]

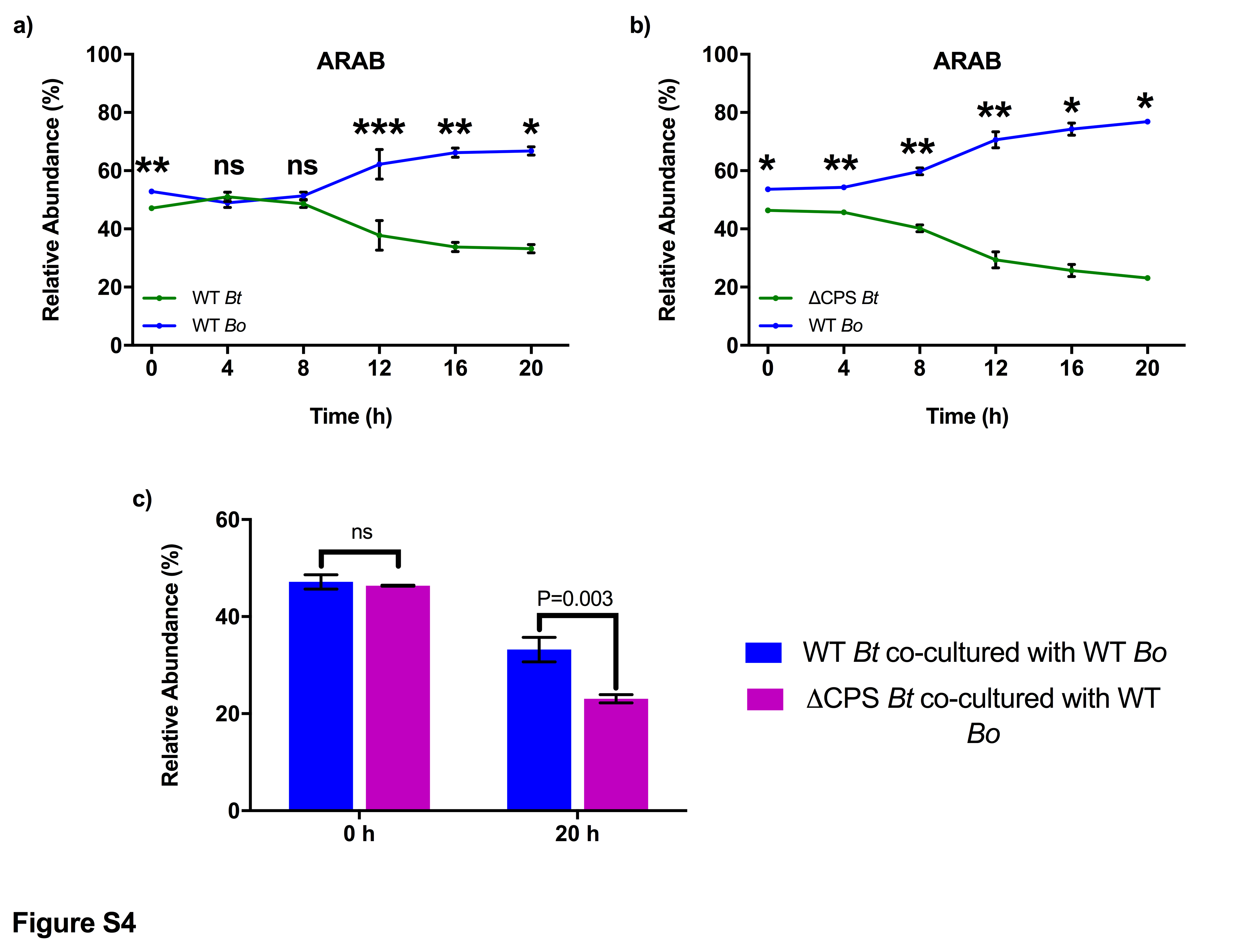

Supplement: FIG S4 [file mbo005173504sf4.tif]

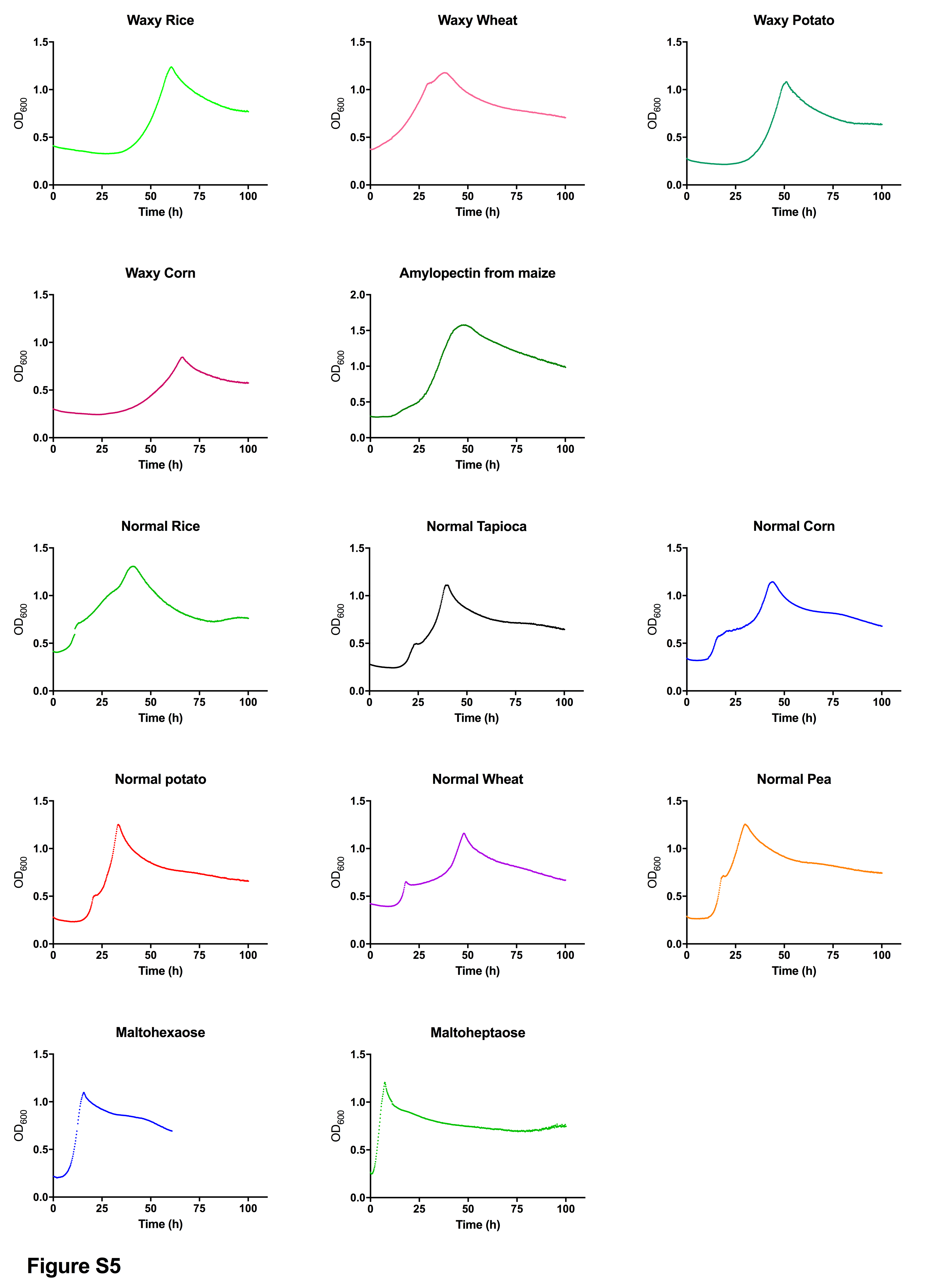

Supplement: FIG S5 [file mbo005173504sf5.tif]

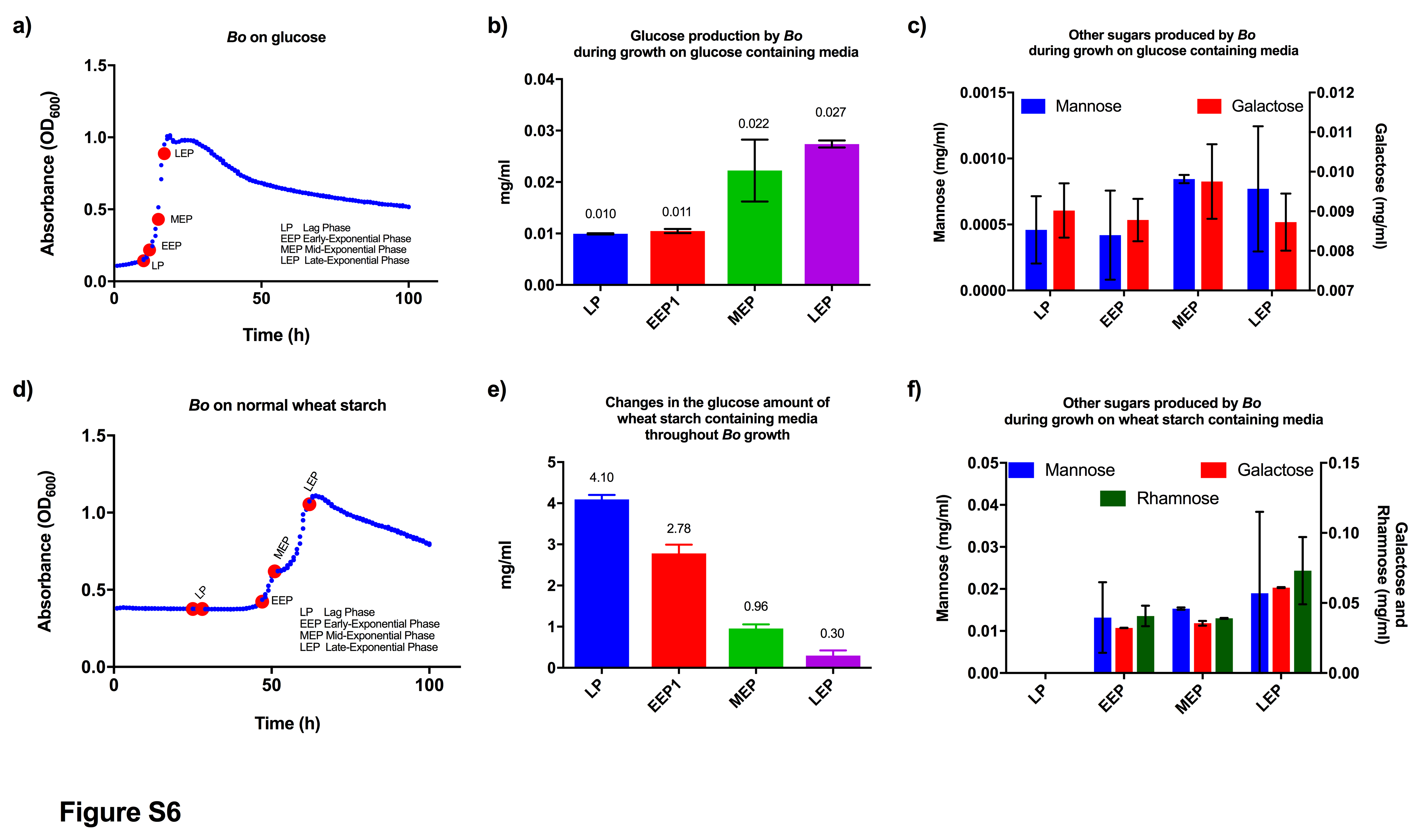

Supplement: FIG S6 [file mbo005173504sf6.tif]

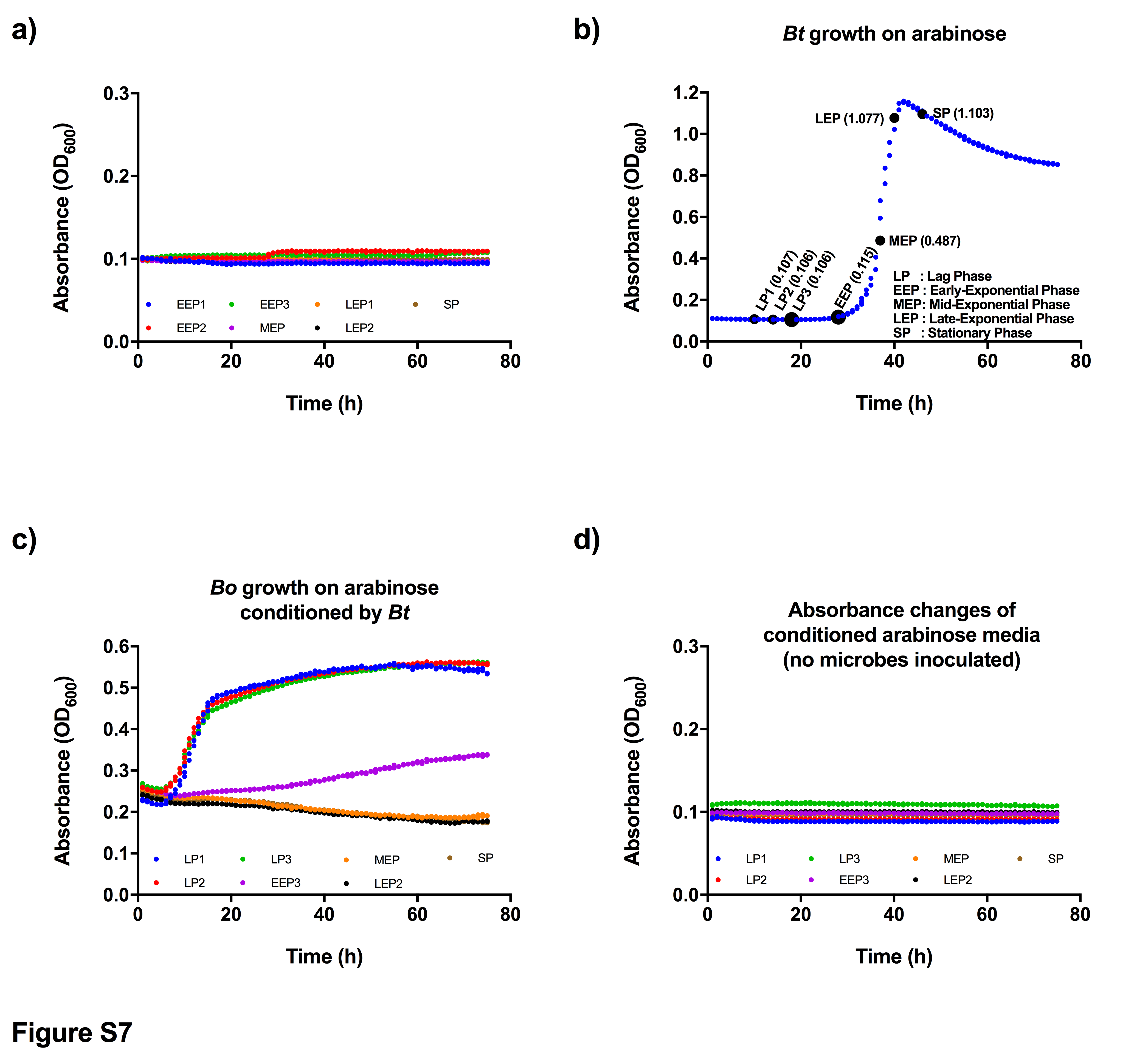

Supplement: FIG S7 [file mbo005173504sf7.tif]
