# Supplementary material for: Reciprocal Prioritization to Dietary Glycans by Gut Bacteria in a Competitive Environment Promotes Stable Coexistence
Source: mBio. 2017 Oct 10;8(5):e01068-17. doi: 10.1128/mBio.01068-17 (PMC5635687; doi:10.1128/mBio.01068-17)
Supplement: TABLE S1 [file mbo005173504st1.docx]

**^1^** Samples with the same letter on the same column are not significantly different (P < 0.01). nd: not determined.

**^2^** Actual growth curves of *Bo* on each substrate are given in Fig. S5.

**^3^** Lag time is equal to T_min_.

**^4^** Density were calculated with the following formula: (OD_max_ - OD_min_).

^5^ Growth rates were calculated with following formula: [(OD_max_ - OD_min_) / (T_max_ - T_min_)]; where, OD_max_ is the point at which OD_600_ reached its maximum; OD_min_ is the minimum OD_600_ measured; T_max_ and T_min_ are the corresponding time values for each absorbance.

**^6^** LSD: Least Significant Difference. **^§^** Molecular sizes of maltohexaose and maltoheptaose are provided by the manufacturer.
