# Supplementary material for: Reciprocal Prioritization to Dietary Glycans by Gut Bacteria in a Competitive Environment Promotes Stable Coexistence
Source: mBio. 2017 Oct 10;8(5):e01068-17. doi: 10.1128/mBio.01068-17 (PMC5635687; doi:10.1128/mBio.01068-17)
Supplement: TEXT S1 [file mbo005173504s1.docx]

**Supplementary Information**

**Results**

***Bt*’s capsular polysaccharides provided a competitive advantage to *Bt***

We had also considered that *Bo* might utilize the capsular polysaccharides of *Bt*, when there was no other glycan present that it could utilize (i.e., when *Bo* was grown in media with ARAB), and this might be the reason why we observed *Bo* started to outcompete *Bt* in competition experiments (**Fig. 3 and 4**). To test this possibility, we co-cultured *Bo* with a capsular polysaccharide-deficient mutant (ΔCPS) of *Bt* in a medium containing ARAB as the only carbon source (in parallel, as a control, wild type *Bt* was co-cultured with *Bo* in a medium containing the same glycan). In both cases, *Bo* outcompeted *Bt* (**Fig. S4a and b**) indicating that deficiency in capsular polysaccharides does not completely rescue *Bt* competitive growth. However, the relative abundance of wild type *Bt* (33.2%) was significantly higher (two tailed student’s t-test, P < 0.005) than that of the isogenic ΔCPS mutant at the end of co-culture (23.1%) (**Fig. S4c**). This suggests that capsular polysaccharides do provide a competitive advantage to *Bt* in co-culture and thus multiple factors including capsule likely influence competitive fitness.

**Carbohydrate synthesis by *Bo* (negative control for Fig. 8)**

In order to test whether *Bo* synthesized any carbohydrate that could interfere with NMR analysis for degree of branching, *Bo* was grown on media containing either glucose or wheat starch, and the cultures were harvested at different growth stages (**Fig. S6a and d**). The media was analyzed for the carbohydrate content. The results revealed that *Bo* synthesized glucose-containing compounds (possibly glycogen or capsular polysaccharides) while growing on glucose, but the amount (**Fig. S6b**) was negligible when compared to the glucose amount found in the media harvested while growing on wheat starch (**Fig. S6e**). In addition to glucose, mannose and galactose were detected in the media, which may be components of its capsular polysaccharides (**Fig. S6c and f**). Interestingly, the amount of mannose and galactose found in the media collected while *Bo* was grown on wheat starch was at least 10 times higher than when it was grown on glucose. More strikingly, the type of monosaccharides found in the harvested media was dependent on the type of substrate on which *Bo* grew; i.e., *Bo* grown on wheat starch had rhamnose, whereas *Bo* did not when grown on glucose.

**Materials and Methods**

***Starches used***

Maltohexaose [product code (PC): M1024] and maltoheptaose (PC: M1025) were purchased from TCI America (TCI America Chemicals, Portland, OR). Potato, pea, and corn starches were gifted by Roquette America, Inc. (Gurnee, IL). Rice and wheat starches were purchased from Sigma-Aldrich (Sigma-Aldrich, St. Louis, MO). Waxy potato and tapioca starches were gifted by Ingredion Inc. (Bridgewater, NJ). Waxy corn starch was gifted by Tate & Lyle Inc., (Hoffman Estates, IL). Waxy rice starch was obtained from Dr. Pinthip Rumpagaporn from the Department of Food Science and Technology, Kasetsart University (Bangkok, Thailand). Waxy wheat starch was extracted from waxy wheat varieties provided by Dr. Craig F. Morris of the Western Wheat Quality Laboratory (Ref # 07-034-0006) (Pullman, WA) as described previously (40).

***Determination of structural features of starch samples***

*Degree of branching:* ^1^H NMR spectroscopy was used to determine the degree of branching (DB) of waxy starch samples as previously described (41) with some modifications. Starch samples (5 mg) were dissolved in 750 μl *d_6_*-DMSO (Sigma, PC:156914) containing 0.5 % lithium bromide (Sigma, PC: 213225). The mixture was transfer to a 5 mm NMR tube using a Pasteur glass pipet. Immediately before acquisition of the NMR spectra, 50 μl of *d_1_*-TFA (Sigma, PC: 152005) was added to the waxy starch samples. ^1^H NMR spectra of samples were measured at 340 K on a Bruker Avance NMR spectrometer (Bruker Biospin, Rheinstetten, Germany), equipped with a TXI probe, operating at a frequency 800.13 MHz with an approximately 8 μs, 90^o^ pulse, repetition time of 9.15 s (composed of an acquisition time of 3.15 s and a relaxation delay of 6 s) and number of scans of 32. DB of samples were calculated using the following formula as previously described (42)

$$DB\left( \% \right)=\frac{I_{\alpha-(1,6)}}{I_{\alpha-(1,6)}+ I_{\alpha-(1,4)}}$$

[I_α-(1,6)_ is the integral of α-(1,6) linkages, and I_α-(1,4)_ is the integral of α-(1,4) linkages].

Prior to DB determination, samples collected in time course assays for DB determination were dialyzed (cutoff = 1 kDa, Spectrum Laboratories, Rancho Dominguez, CA) against purified water for at least 36 h to remove vitamins and minerals from the minimal media, followed by lyophilization.

*Amylose and amylopectin content:* Amylose and amylopectin contents of waxy starch samples were calorimetrically determined in triplicate using an amylose/amylopectin assay kit (Megazyme, PC: K-AMYL) according to manufacturer’s instructions (Megazyme International, Wicklow, Ireland).

*Molecular size distribution:* Sample preparations were carried out as previously described (43). Molecular size distribution of waxy starch samples was determined using a high-performance size exclusion chromatograph equipped with multi-laser scattering and refractive index detectors (HPSEC-MALS-RI) [a DAWN DSP-F laser photometer fitted with argon laser at λ = 488 nm with a K-5-129 flow cell (Wyatt Technology, Santa Barbara, CA) and an Optilab 903 interferometric refractometer (Wyatt Technology, Santa Barbara, CA)], a pump (model LC-10AT vp, Shimadzu Corp., Columbia, MD), and syringe sample loading injector (model 7125, Rheodyne Inc., Catati, CA) as previously reported (42). A *dn/dc* value of 0.146 was used in molecular size calculations and data processing was done using ASTRA software (Version 4.9, Wyatt Technology, Santa Barbara, CA).

***Sample collection and measurement of the remaining glycans in the experiment where the effects of glycan structure on its prioritization by Bo were studied (for the experiment of Fig. 7).***

Two aliquots were sampled every hour during *Bo* growth on glycan mixture (**Fig. 6**); one of which was for measuring the remaining glycans in the media (2 ml), and the other for monitoring PUL expression over time relative to time 0 (1 ml).

Collected samples for glycan analysis (0.5 ml) were lyophilized. Neutral monosaccharides found in these samples were determined as their alditol acetate derivatives (44) using gas chromatography coupled with mass spectrometry (7890A and 5975C inert MSD with a Triple-Axis detector, Agilent Technologies, Inc., Santa Clara, CA) (GC/MS). Prior to preparing trimethylsilyl (TMS) derivatives, the remaining 1.5 ml of collected samples for glycan analysis were dialyzed (cutoff = 1 kDa, Spectrum Laboratories, Rancho Dominguez, CA) against purified water for at least 36 h. This was because preliminary analysis showed that presence of vitamins and minerals that come from minimal media, as well as compounds possibly produced by bacteria species during growth on the glycan mixture, interfere with TMS derivatization and gas chromatography analysis (data not shown). Dialyzed samples were then lyophilized. TMS derivatives of these samples were prepared as described previously (45) and analyzed on a gas chromatograph with a DB-5 capillary column (Agilent Technologies, Santa Clara, CA) to determine the acidic monosaccharide and rhamnose content of the samples on a weight basis. Even though the method of TMS derivatives of sugars is suitable for simultaneous determination of neutral and acidic sugars, this was not used because maltohexaose used in one of the time-course assays has a molecular weight of 942 Da (**Table S1**) and it would be removed during dialysis. Glycosyl-linkage profiles of the dialyzed samples were carried out as their partially methylated alditol acetate derivatives using GC/MS (44).

Over the time course, the disappearance percentages of AP (or MH) and PG relative to their initial amounts were calculated by quantifying the glucose and galactose amounts in the samples as their alditol acetates, respectively. Similarly, the remaining ARAB amount was quantified by measuring the remaining 5-arabinose linkage over time. Residual PGA amount was determined by subtracting total rhamnose from total galacturonic acid amount calculated as their TMS derivatives. Remaining CS amounts were determined by quantifying the remaining glucuronic acid amounts as their TMS derivatives. These residues are signatures of each corresponding glycan and the measured glycosyl residues were used to calculate the amount remaining of the corresponding glycans.

***Preparation of ARAB containing media conditioned by Bt and Bo growth on these media***

*Bt* was grown on ARAB-containing media (initial ARAB concentration, 5 mg/ml). Throughout its growth, media were harvested at different time points (**Fig. 5b**), and then centrifuged at 10,000 rpm for 10 min, and supernatants were filter-sterilized (0.22 μ pore size).

The growth of *Bo* on the filter-sterilized media were monitored using a custom carbohydrate array constructed in a 96-well format using an automated plate reader as described in the text. Filter-sterilized media (100 μl) were placed into each well of a 96-well plate. *Bo* culture for inoculation was pre-grown to mid-exponential phase (OD_600_ 0.5 – 0.7) on minimal media (MM) containing glucose, and 10 ml aliquots were centrifuged to pellet cells, which were then washed with MM containing no carbon source. These washed cells were used to inoculate 10 ml of MM containing no carbon source. Cell suspensions (100 μl) were inoculated in wells containing 100 μl of filter-sterilized media to obtain 200 μl cultures. Plates were sealed and loaded into an automated plate reader as described in the text. As a negative control, 200 μl aliquots containing equal volume of filter-sterilized media and MM (containing no bacterial cells) were also placed into the plates (**Fig. S7a**). As a positive control, growth of *Bo* was monitored on arabinose-containing media conditioned by *Bt* (**Fig. S7c**). The arabinose-containing medium predigested by *Bt* at different growth stages was obtained by harvesting cultures throughout *Bt* growth in medium containing arabinose as the only carbon source (**Fig. S7b**), which were filter-sterilized before inoculation with *Bo* as noted above.

***Thin-layer chromatography (TLC) analysis***

Thin-layer chromatography was performed using 20 cm x 20 cm glass silica gel plates (Millipore, PC:105715, EMD Millipore, Billerica, MA). Filter-sterilized samples (3 μl) were spotted on the plate. The plates were developed in a TLC tank using solution containing chloroform, acetic acid, and water at ratio of 6, 7, and 1, respectively. After development, the plates were air-dried. They were visualized by washing with a staining solution [sulfuric acid/methanol (5:95, v/v) containing 0.03 mg/ml N-(1-naphtyl) ethylenediamine dihydrochloride (Sigma-Aldrich, PC: 222488)], followed by heating at 110 – 120 ^o^C for 5 min. Arabinooligosaccharide standards for TLC [arabinan (PC: P-ARAB), arabinobiose (PC: O-ABI), arabinohexaose (PC: O-AHE), and arabinoheptaose (PC: O-AHP)] were purchased from Megazyme (Megazyme International, Wicklow, Ireland). Arabinose (PC: A3131) was purchased from Sigma-Aldrich. Before running on TLC plates, standards were also mixed with MM-containing no carbon source at a ratio of 1:1.

**References**

40. Reddy I, Seib PA. 2000. Modified waxy wheat starch compared to modified waxy corn starch. Journal of Cereal Science 31:25-39.

41. Tizzotti MJ, Sweedman MC, Tang D, Schaefer C, Gilbert RG. 2011. New H-1 NMR procedure for the characterization of native and modified food-grade starches. Journal of Agricultural and Food Chemistry 59:6913-6919.

42. Zhang B, Dhital S, Flanagan BM, Gidley MJ. 2014. Mechanism for starch granule ghost formation deduced from structural and enzyme digestion properties. Journal of Agricultural and Food Chemistry 62:760-771.

43. Zhang G, Ao Z, Hamaker BR. 2006. Slow digestion property of native cereal starches. Biomacromolecules 7:3252-3258.

44. Pettolino FA, Walsh C, Fincher GB, Bacic A. 2012. Determining the polysaccharide composition of plant cell walls. Nature Protocols 7:1590-1607.

45. Doco T, O'Neill MA, Pellerin P. 2001. Determination of the neutral and acidic glycosyl-residue compositions of plant polysaccharides by GC-EI-MS analysis of the trimethylsilyl methyl glycoside derivatives. Carbohydrate Polymers 46:249-259.
