# Supplementary material for: Reciprocal Prioritization to Dietary Glycans by Gut Bacteria in a Competitive Environment Promotes Stable Coexistence
Source: mBio. 2017 Oct 10;8(5):e01068-17. doi: 10.1128/mBio.01068-17 (PMC5635687; doi:10.1128/mBio.01068-17)
Supplement: TABLE S2 [file mbo005173504st2.docx]

**Table S2.** Primers used in this study

| **Primer Name** | **Sequence (written 5' to 3')** | **Use** |
| --- | --- | --- |
| ***Bo*** |  |  |
| BACOVA01998 F | CACTCGGGATGGCAGGAATG | CS PUL expression |
| BACOVA01998 R | CAATGAACCGGAAGGGAAATAGC | CS PUL expression |
| BACOVA04911 F | CAAAGCTTTAGCCAACCAAT | PGA PUL expression |
| BACOVA04911 R | ACCGTCTGCACGTACTGTAA | PGA PUL expression |
| BACOVA05491 F | ATCCGTGTGCGTGGTGGTG | PG PUL expression |
| BACOVA05491 R | TTCGGGTTGATGAGGCTAAGGA | PG PUL expression |
| BACOVA05007 F | TCCAGCCTGTCCGACCCTACTAT | RGI PUL expression |
| BACOVA05007 R | CCCCAAACGTTCGGACTTACC | RGI PUL expression |
| BACOVA03518 F | CGTTATACCGCCTCCGTCAATC | AP PUL expression |
| BACOVA03518 R | ATAGCCCCGTCGTCAGCATAAC | AP PUL expression |
| 16S F | TTTGTTGGCGGGGTAACGGCC | qPCR normalization |
| 16S R | CTTGGCTGGTTCAGGCTCTCG | qPCR normalization |
| BACOVA03426 F | CCTGCCAATGCGGAAGAATG | Enumeration - specific to *Bo* |
| BACOVA03426 R | AGCCGCTGCAAAATAACTGACAA | Enumeration - specific to *Bo* |
| ***Bt*** | | |
| BT3332F | TGTTCCCGGAGCCAGTGTTC | CS PUL expression |
| BT3332R | TTCGTCCAGCGTTTTAGTATCTTCTTT | CS PUL expression |
| BT4114F | CGCAACGGAAGCACTAACAGG | PGA PUL expression |
| BT4114R | GGGAAGCCGTCTACAATAAATAAA | PGA PUL expression |
| BT4671F | ATGGTTTCATCGCCCGAAGAG | PG PUL expression |
| BT4671R | GCGTATGGTTGAGACAGATGTAGG | PG PUL expression |
| BT4164F | GAAATGTAATGAATGATGCAAAAGGTAGA | RGI PUL expression |
| BT4164R | CGAAACGTCCGTGGAAGAAAGTA | RGI PUL expression |
| BT3702F | GCTATTGGCGGGGCATTGG | AP PUL expression |
| BT3702R | CAGCGGATTTTGGGGAGAGTTCG | AP PUL expression |
| BT0364F | TGAATGGCGGTAAGGTAAAAGAACA | ARAB PUL expression |
| BT0364R | CGGGCCGGAAGCGAGTAG | ARAB PUL expression |
| 16S F | ATGGTATAATCAGACCGCATGG | qPCR normalization |
| 16S R | GAGCCGTTACCTCACCAACTGC | qPCR normalization |
